# Heme and hemoglobin utilization by *Mycobacterium tuberculosis*

**DOI:** 10.1038/s41467-019-12109-5

**Published:** 2019-09-18

**Authors:** Avishek Mitra, Ying-Hui Ko, Gino Cingolani, Michael Niederweis

**Affiliations:** 1 Department of Microbiology, University of Alabama at Birmingham, 845 19th Street South, Birmingham, AL 35294 USA; 2 Department of Biochemistry and Molecular Biology, Thomas Jefferson University, 233S. 10th Street, Philadelphia, PA 19107 USA; 3 Institute of Biomembranes and Bioenergetics, National Research Council, Via Amendola 165/A, 70126 Bari, Italy

**Keywords:** Metalloproteins, Bacterial structural biology, Pathogens

## Abstract

Iron is essential for growth of *Mycobacterium tuberculosis* (Mtb), but most iron in the human body is stored in heme within hemoglobin. Here, we demonstrate that the substrate-binding protein DppA of the inner membrane Dpp transporter is required for heme and hemoglobin utilization by Mtb. The 1.27 Å crystal structure of DppA shows a tetrapeptide bound in the protein core and a large solvent-exposed crevice for heme binding. Mutation of arginine 179 in this cleft eliminates heme binding to DppA and prevents heme utilization by Mtb. The outer membrane proteins PPE36 and PPE62 are also required for heme and hemoglobin utilization, indicating that these pathways converge at the cell surface of Mtb. Albumin, the most abundant blood protein, binds heme specifically and bypasses the requirements for PPE36, PPE62 and Dpp. Thus, our study reveals albumin-dependent and -independent heme uptake pathways, highlighting the importance of iron acquisition from heme for Mtb.

## Abstract (web summary)

Iron is essential for growth of *Mycobacterium tuberculosis*, but most of the iron in the human body is stored in heme within hemoglobin. Here, Mitra et al. identify two heme uptake mechanisms in *M. tuberculosis*, one dependent on the inner-membrane Dpp importer and the other dependent on host albumin.

## Introduction

Iron, due to its versatile coordination properties and tunable redox state, is used in many essential biological processes^1^. An important part of the innate immune response to bacterial infections is the limitation of accessible iron in the human host which is sequestered in high-affinity binding proteins such as transferrin, ferritin, and lactoferrin or in heme^2^. Hence, for many bacterial pathogens, iron is the limiting factor for colonization and infection^3^. A large body of evidence links iron availability to tuberculosis (TB) pathogenesis^4^. For example, iron overload was found to increase the risk of TB, of TB treatment failure, and of mortality in TB patients. Human genetic disorders or gene variations such as haptoglobin and NRAMP1 polymorphisms increased iron levels in macrophages and the risk of TB and/or worsen disease outcomes. It was concluded that the host’s iron status is an important yet under-evaluated factor in TB prevention and therapy^4^.

Recently, Rodriguez and co-workers^5^ showed that the necrotic centers of granulomas of human TB patients contain a high concentration of host iron-sequestering proteins and iron-restricting factors, likely establishing an iron-deprived environment for *M. tuberculosis* (Mtb). These results not only highlighted strict iron restriction at the primary sites of Mtb infection in humans but, unexpectedly, also revealed that prolonged iron starvation triggers a transition of Mtb to a persistent state, a hallmark of chronic TB^5^. To counteract the iron limitation in the host, Mtb secretes siderophores, small molecules with extremely high iron-binding affinities, called (carboxy)mycobactins^6–8^. Mtb can also directly utilize heme and hemoglobin as iron sources^9,10^. The importance of these iron acquisition mechanisms for virulence of Mtb was shown in several reports. For example, the ATP-binding cassette (ABC) transporter IrtAB is required for efficient utilization of iron from ferric carboxymycobactin and for replication of Mtb in macrophages and mice^11^. A mutant of Mtb lacking ferritin, an iron storage protein, is unable to establish a chronic infection in mice^12^ and IdeR, the main regulator of iron homeostasis in Mtb, is required for virulence in Mtb^13^.

It is estimated that more than 70% of iron in the human body is tightly bound in heme and complexed with hemoglobin making heme the major iron source in the human host^14^. In diderm bacteria host hemoproteins or heme are captured by outer membrane receptors to transport heme into the periplasm. Heme is then transported across the inner membrane by permeases, which are specific ABC importers^14^. Recently, we identified PPE36 and PPE62 as membrane-anchored, heme-binding proteins on the cell surface of Mtb that are required for heme utilization^15^. PPE36 and PPE62 do not share any sequence similarity with known heme-binding proteins^16^. MmpL3 and MmpL11 were proposed as the inner membrane importers of heme in Mtb^10^. However, MmpL3 and MmpL11 are RND-type efflux pumps involved in export of trehalose monomycolate, lipids, or other lipid-like molecules for maintenance of the mycobacterial cell wall^17–19^. Thus, the roles of MmpL3 and MmpL11 in heme utilization by Mtb are unclear^15^.

Herein, we show that heme and hemoglobin utilization converge at the cell surface of Mtb after heme is released from hemoglobin and demonstrate that the substrate-binding protein DppA is required for heme uptake across the inner membrane of Mtb. We also show that Mtb is capable of utilizing heme in the presence of albumin bypassing the requirement of PPE and DppA proteins.

## Results

### The Dpp transporter is essential for heme utilization by Mtb

**Fig. 1:**
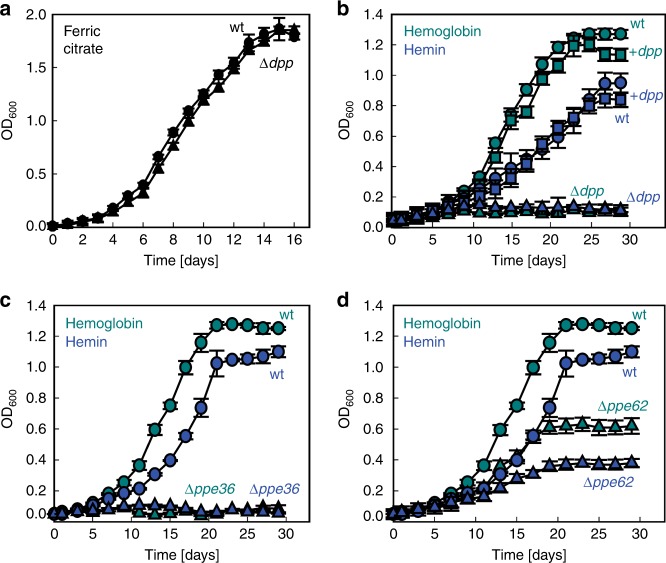
Heme and hemoglobin utilization by *M. tuberculosis* and mutant strains. Growth of the avirulent Mtb strain mc^2^6206 (wt; circles), the Δ*dpp* mutant ML2436 (triangles), and the complemented Δ*dpp* mutant (squares) in HdB minimal medium with **a** 10 µM ammonium ferric citrate and **b** 2.5 µM human hemoglobin (cyan) or 10 µM hemin (blue). Growth of Mtb mc^2^6206 (circles), the Δ*ppe36* mutant ML2411 (triangles) (**c**), and the Δ*ppe62* mutant ML2412 (triangles) (**d**) in HdB minimal medium with 2.5 µM hemoglobin (cyan) or 10 µM hemin (blue). Medium with hemin and hemoglobin contained 20 µM of 2,2′-dipyridyl to prevent utilization of trace ferric iron. Error bars represent standard errors of mean values of biological triplicates. Source data are provided in the Source Data file

To identify potential inner membrane heme uptake systems, we searched for Mtb proteins with similarities to bacterial heme transporters. While Mtb does not have any homologs of the heme permease HemTUV^14,20^ (Supplementary Table 1), the Mtb *rv3666c-rv3663c* operon encodes four proteins that share ~24–45% sequence similarities with the dipeptide transporter Dpp of *Escherichia coli*, which also enables heme uptake^21^. The components of the Mtb Dpp transporter include DppA (R3666c; substrate-binding protein), DppB/C (R3665c/Rv3664c, integral membrane permease proteins), and DppD (Rv3663c, ATPase). To examine whether the Mtb Dpp transporter is involved in heme utilization, we deleted the entire *dpp* operon in the avirulent Mtb strain mc^2^6206 by homologous recombination as previously described^15^. The Mtb Δ*dpp* mutant was named ML2436 (Supplementary Fig. 1B, C; Supplementary Table 2). Deletion of the *dpp* operon did not affect the growth of Mtb in HdB minimal medium with 10 µM ammonium ferric citrate as the sole iron source (Fig. 1a), but completely abolished growth with 10 µM hemin and 2.5 µM human hemoglobin (Fig. 1b). The initial residual growth of the Mtb Δ*dpp* mutant is probably due to incomplete depletion of the internal iron storage^12,22^. The *dpp* operon expression vector pML3757 (Supplementary Table 3) restored the ability of the *Δdpp* mutant to utilize hemin and hemoglobin demonstrating that the growth defect of Mtb ML2436 was indeed caused by the lack of the *dpp* operon (Fig. 1b, Supplementary Fig. 2). These results show that the Dpp transporter is essential under these growth conditions for both heme and hemoglobin utilization in Mtb.

The Mtb genes *rv1283c*-*rv1280c*, annotated as *opp* genes, encode proteins that share sequence similarities with bacterial oligopeptide and dipeptide transporters^23^. However, the *rv1283c*-*rv1280c* operon expression plasmid pML3758 (Supplementary Table 3) did not restore heme utilization in the *Δdpp* mutant ML2436 in contrast to the *dpp* operon expression vector (Supplementary Fig. 2). This result is consistent with the severe growth defect of the Mtb *Δdpp* mutant, despite the presence of a functional *opp* operon, in medium with heme as the only iron source (Fig. 1b). Collectively, these results show that the Dpp transporter is the major heme transporter in the inner membrane of Mtb.

### PPE proteins are required for heme and hemoglobin utilization by Mtb

Previously, we identified PPE36 and PPE62 as membrane-anchored cell surface-accessible proteins which bind heme and are required for efficient heme utilization of Mtb^15^. Here, we sought to examine whether PPE36 and PPE62 also play a role in hemoglobin utilization by Mtb. The *Δppe36* mutant ML2411 did not grow in HdB minimal medium with human hemoglobin or with heme as the sole iron source (Fig. 1c) demonstrating that PPE36 is essential for both heme and hemoglobin utilization by Mtb under these growth conditions. Growth of the *Δppe62* mutant ML2412 with heme and hemoglobin was also impaired (Fig. 1d) demonstrating that PPE62 plays an important role both in heme and hemoglobin utilization. This result also indicated that PPE62 has a functional homolog in Mtb, which accounts for the residual growth of the *Δppe62* mutant.

**Fig. 2:**
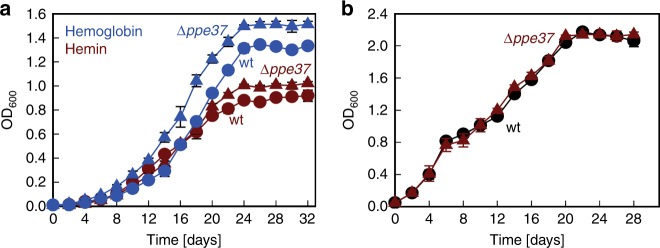
Characterization of the *M. tuberculosis* Δ*ppe37* mutant. **a** Growth of avirulent Mtb strain mc^2^6206 (wt; circles) and Δ*ppe37* mutant (triangles) in HdB minimal medium with 10 µM hemin (red) or 2.5 µM human hemoglobin (blue). **b** Growth of avirulent Mtb strain mc^2^6206 (wt; black circles) and Δ*ppe37* (red triangles) in HdB minimal medium with 0.5% bovine serum albumin and 10 µM hemin. All media contained 20 µM of 2,2′-dipyridyl to prevent utilization of trace iron. Error bars represent standard errors of mean values of biological triplicates. Source data are provided in the Source Data file

A recent study showed that PPE37 (Rv2123) is essential for heme utilization in the Mtb Erdman strain and that deletion of *ppe36* had no effect^24^. This was puzzling considering that the *ppe36* gene is essential for both heme and hemoglobin utilization in the avirulent Mtb strain mc^2^6206 (Fig. 1c) consistent with our previous results^15^. Mtb mc^2^6206 (H37Rv Δ*panCD* Δ*leuCD*) is an isogenic, avirulent Mtb strain^25^ derived from Mtb H37Rv, a laboratory Mtb strain commonly used in research. To address this discrepancy, we deleted the *ppe37* gene in Mtb mc^2^6206 (Supplementary Fig. 3B, Supplementary Table 2). Growth of the Δ*ppe37* mutant in HdB minimal medium in the presence of 10 µM hemin or 2.5 µM human hemoglobin was identical to that of the parent strain confirming that PPE37 is not required for growth on heme in Mtb mc^2^6206 (Fig. 2a). It should be noted that we did not examine whether PPE37 is required for heme utilization in Mtb H37Rv.

### Directionality of heme transport by the Dpp system

**Fig. 3:**
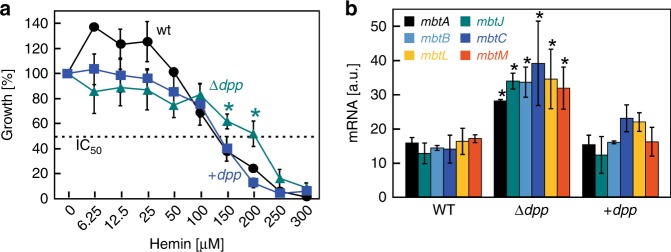
The *M. tuberculosis Δdpp* mutant is resistant to heme toxicity and experiences iron starvation. **a** Growth of wt Mtb (circles), the Δ*dpp* mutant (triangles), and the complemented Δ*dpp* mutant (squares) in HdB minimal medium with 1 µM ammonium ferric citrate and increasing concentrations of hemin. Note that the scale of the *y*-axis is not linear. **b** The mRNA levels of *mbt* genes of wt Mtb, the Δ*dpp* mutant, and the complemented Δ*dpp* mutant in the presence of 10 µM hemin were determined by quantitative real-time PCR. Error bars represent standard errors of mean values of biological triplicates. For all plots, asterisks denote significant differences for Δ*dpp* mutant compared to wt Mtb. Statistical significance was determined by Tukey’s HSD following an *F*-test (*p* < 0.05). Source data are provided in the Source Data file

Many heme-utilizing bacteria have ABC transporter efflux systems for heme detoxification^26,27^. To examine whether the Dpp transporter is involved in heme uptake or efflux, we monitored the growth of wt Mtb, the Δ*dpp* mutant and the complemented strain in HdB minimal medium containing 1 µM ferric citrate and increasing concentrations of hemin. Hemin was toxic for wt Mtb with an IC_50_ of ~135 µM (Fig. 3a). Deletion of the *dpp* genes increased the toxic concentration of hemin to an IC_50_ of ~200 µM. The susceptibility of the Δ*dpp* mutant to hemin was fully restored to wt levels by expression of the *dpp* operon (Fig. 3a). The increased resistance of the Δ*dpp* mutant to heme toxicity strongly suggests that the Dpp transporter is not involved in heme efflux but rather in heme uptake. In an alternative approach to examine whether the Dpp transporter is involved in heme uptake, we determined transcription levels of six mycobactin biosynthesis genes (*mbt*) from two *mbt* loci representing three operons^28^ as an indicator of iron availability^7^. The mRNA levels of all six selected *mbt* genes as determined by quantitative real-time PCR were increased by more than two-fold in the Δ*dpp* mutant compared to wt Mtb when the strains were grown in minimal medium with hemin as the sole iron source (Fig. 3b). Expression of the *dpp* operon reduced transcription of the *mbt* genes to wt levels demonstrating that the iron starvation of the Δ*dpp* mutant was indeed due to the lack of the *dpp* operon (Fig. 3b). The iron starvation of the Δ*dpp* mutant in HdB medium with heme as the sole iron source and its increased resistance to the toxicity of heme strongly indicate that the Dpp transporter of Mtb is involved in heme uptake and not in heme efflux. This conclusion is consistent with the function of the Dpp systems in other bacteria^14,21,29^.

### Cell permeability of Mtb Δ*dpp* to hydrophobic compounds

It was previously reported that deletion of the *rv3665c-rv3663c* genes in a clinical Mtb strain alters levels of the outer membrane lipids phthiocerol dimycocerosates (PDIMs) and mycolic acids^23^. Since heme is a hydrophobic molecule, it is possible that deletion of the *dpp* operon could alter the outer membrane lipid composition and enhance its function as a permeability barrier, thereby reducing heme uptake. To examine the cell permeability of the Δ*dpp* mutant, we measured the uptake of ethidium bromide as a hydrophobic compound whose uptake is detectable by fluorescence^30^. The uptake rates for ethidium bromide by wt Mtb and the Δ*dpp* mutant were identical (Supplementary Fig. 4), indicating that the heme uptake defect in the Δ*dpp* mutant is not due to altered cell permeability.

### Role of peptides in heme uptake by Mtb

Previous studies showed that Dpp-dependent heme utilization in *E. coli* is inhibited by peptides^21^. Deletion of *dpp* genes in a clinical Mtb isolate conferred resistance to the toxic tripeptide bialaphos suggesting that the Mtb Dpp transporter is also involved in peptide transport^23^. To examine whether peptides interfere with heme uptake, we examined the growth of Mtb in HdB minimal medium with ammonium ferric citrate, hemin, or human hemoglobin as iron sources in the presence of peptides. While 1% tryptone, a tryptic digest of the milk protein casein, did not affect growth of Mtb in medium with ammonium ferric citrate, growth with heme or hemoglobin was reduced (Supplementary Fig. 5). Expression of the *dpp* operon (pML3757; Supplementary Table 3) increased growth of Mtb with heme and hemoglobin as iron sources in the presence of tryptone-derived peptides (Supplementary Fig. 5). Collectively, these results indicate that peptides can interfere with heme uptake by the Mtb Dpp transporter.

### DppA is a heme-binding protein of Mtb

**Fig. 4:**
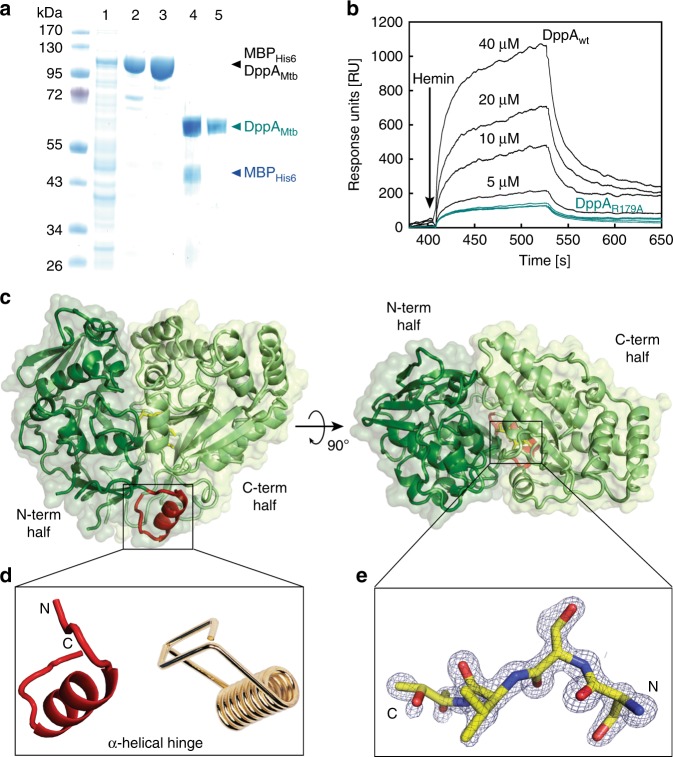
Heme binding by *M. tuberculosis* DppA and its atomic structure. **a** Purification of DppA from *E. coli*. Lanes: (1) *E. coli* lysate containing *mbp*_*6his*_*-dppA* expression vector, combined fractions after Ni(II)-affinity (2) and amylose affinity (3), (4) TEV cleavage of fusion protein and (5) purified DppA post TEV cleavage after Ni(II) recapture of MBP_6His_. **b** Heme binding of wild-type DppA_Mtb_ (black) and mutant DppA_R179A_ (cyan) at varying heme concentrations determined by surface plasmon resonance (SPR) spectroscopy. Buffer controls do not show any signal for both proteins and are not visible because they overlap with the *x*-axis. The same heme concentrations were used for both proteins. Source data are provided in the Source Data file. **c** Ribbon diagram of Mtb DppA with N- and C-terminal halves of the protein color-coded in dark and light green, respectively. The ribbon diagram is overlaid to a semi-transparent solvent surface. **d** Magnified view of DppA α-helical hinge (residues 250–266) colored in red, which bears a striking resemblance to a clothespin spring. **e** Magnified view of DppA_wt_ final 2Fo−Fc electron density map calculated at 1.27 Å resolution and overlaid to the refined model of DppA tetrapeptide (shown as sticks). The electron density is colored in blue and contoured at 1.7*σ* above background

Bacterial DppA proteins are periplasmic proteins with a classical Sec signal peptide that have been implicated in heme binding^21^. To examine whether DppA of Mtb (Rv3666c) binds heme, we constructed an expression vector for *E. coli* encoding DppA_Mtb_ lacking the predicted signal peptide and produced milligram quantities of apparently pure recombinant DppA_Mtb_ (Fig. 4a). Addition of hemin to DppA_Mtb_ in surface plasmon resonance experiments resulted in a dose-dependent increase in signal intensity indicative of heme binding (Fig. 4b). The dissociation constant *K*_d_ was calculated to be ~1.5 µM. Thus, the heme affinity for DppA_Mtb_ is higher than that of other DppA proteins with dissociation constants ranging from ~10 µM for DppA_Ec_ to ~655 µM for the DppA homolog HbpA of *Haemophilus influenzae* (Supplementary Table 4). We also examined heme binding of DppA_Mtb_ by difference absorption spectroscopy. Subtracting the free heme spectra from the heme-protein spectra resulted in a characteristic Soret peak at ~410 nm and a broad Q band beak at ~550 nm (Fig. 6b, see below) which are indicative of heme binding by DppA_Mtb_ in solution. These results demonstrate that DppA_Mtb_ is a heme-binding protein.

### High-resolution crystal structure of Mtb DppA

We obtained high-quality crystals of Mtb DppA that were used to determine a 3D-structure of the protein at 1.27 Å resolution. The atomic model of DppA, refined to an *R*_work/free_ of 12.8/16.5% (Table 1), sheds light on the molecular architecture of this periplasmic heme carrier with high accuracy. Mtb DppA has a globular shape that resembles a heart (Fig. 4c). Despite the compact appearance, the DppA tertiary structure is built by two globular and slightly offset halves that fold onto each other with perfect complementarity like the shells of a clam. The N-terminal half of the molecule (residues 1–249) connects to the C-terminal half (residues 267–541) by a flexible α-helical hinge (residues 250–266) that has higher than average B-factor in our structure (28.0 vs. 18.6 Å^2^). This hinge resembles a “clothespin spring” that keeps the two halves of DppA in a closed conformation poised to open up (Fig. 4d). Mtb DppA is similar to the homologous proteins found in Gram-negative bacteria, especially to the *Salmonella typhimurium* ortholog that superimposes to Mtb DppA with an RMSD ~1.45 Å. At the interface between the two DppA halves, completely buried inside the protein core, is a tetrapeptide whose amino acid sequence was modeled as Ser-Ser-Val-Thr (Fig. 4e). The electron density for this peptide is well-resolved, consistent with a tight binding. Accordingly, all attempts to remove the tetrapeptide from DppA by dialysis against 1 M urea followed by re-crystallization failed.

**Table 1:** Crystallographic data collection and refinement statistics

	DppA_wt_	DppA_R179A_
**Data collection**		
Space group	P2_1_	P2_1_
Cell dimensions		
* a*, *b*, *c* (Å)	52.7, 69.1, 62.4	52.6, 68.9, 62.1
* α, β, γ* (°)	90.0, 106.5, 90.0	90.0, 103.3, 90.0
Resolution (Å)	50–1.27 (1.32–1.27)^a^	15–1.25 (1.29–1.25)^a^
*R*_sym_	6.6 (50.7)	8.9 (72.3)
*I*/*σI*	37.8 (2.3)	30.4 (2.2)
Completeness (%)	91.1 (51.3)	98.7 (89.6)
Redundancy	6.1 (3.2)	4.2 (3.4)
**Refinement**		
Resolution (Å)	50–1.27	15–1.25
No. of reflections	98,354	114,872
*R*_work_/*R*_free_^a^	12.8/16.5	16.8/18.8
No. of atoms		
Protein	4389	4402
Water	590	619
*B*-factors		
Protein	21.7	19.5
Water	30.4	27.3
R.m.s. deviations		
Bond lengths (Å)	0.009	0.005
Bond angles (°)	1.441	0.789

^a^Values in parentheses are for highest-resolution shells

### Identification of a potential heme-binding site by modeling

**Fig. 5:**
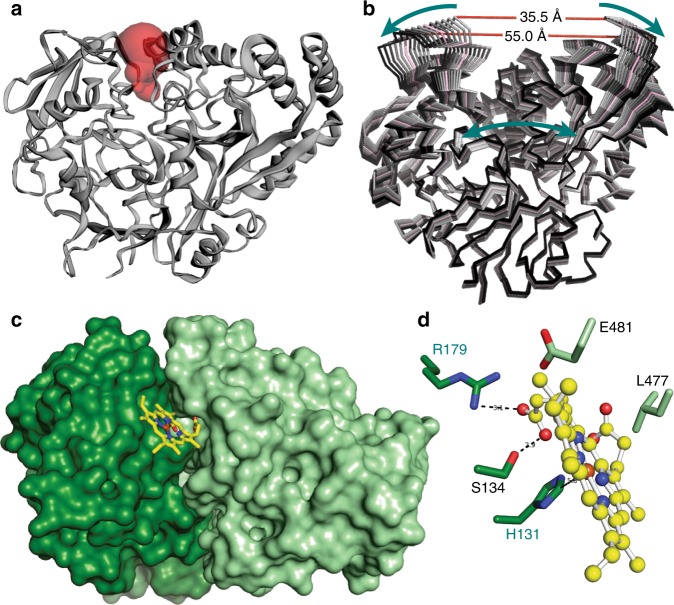
Structure-based identification of a heme-binding pocket in *M. tuberculosis* DppA. **a** Ribbon diagram of DppA overlaid with the volume of the solvent-accessible pocket identified by CASTp. The pocket has a Richards’ solvent-accessible surface area of 333.4 Å^2^ and solvent-accessible volume ~268.4 Å^3^. **b** Superimposition of the 10 perturbed conformations of DppA obtained by applying normal mode analysis using three lowest frequency normal modes. The arrows show the direction of opening of the two N- and C-terminal halves. The crystallographic model of DppA_wt_ is colored in red, while all other states are ramped from light gray (most closed) to black (most open). **c** Docking model of heme (in yellow sticks) bound to the open conformation of DppA (shown as solvent surface with N- and C-terminal halves color-coded as in **a**). **d** Zoom-view of DppA residues making contact with heme

To visualize how DppA binds heme, we formed a complex of DppA with heme in solution and purified the complex by size exclusion chromatography (Supplementary Fig. 6A). The presence of the DppA–heme complex was indicated by the reddish-color of DppA-containing fractions (Supplementary Fig. 6B, C). Absorption spectroscopy of the pooled fractions revealed a strong Soret peak at 405 nm characteristic for heme-bound proteins (Supplementary Fig. 6D). We obtained at least three morphologically different crystals of the purified DppA–heme complex in buffers of high and low ionic strengths and pH values ranging from pH 4.5 to 8 and determined their atomic structures. All crystals shared an identical primitive monoclinic cell with only 34% solvent content (Table 1). Surprisingly, none of these crystallized DppA proteins were bound by heme. We hypothesized that the effect of dehydrating agents used for crystallization and crystal lattice forces stabilize a closed conformation of DppA incompatible with heme binding. To test this hypothesis, we scanned the DppA surface for solvent-accessible pockets that could be filled by heme and expose at least one histidine, the major iron-binding residue in proteins^14^. The CASTp software^31^ identified a solvent-accessible pocket at the interface generated by N- and C-terminal halves with a Richards’ solvent-accessible volume of ~268 Å^3^ (Fig. 5a). This cleft contains several putative heme-binding residues, including His131 and Arg179, and is filled with solvent in our structure. However, it is too small to accommodate heme, as also suggested by in silico docking experiments that failed to yield a convincing solution of a DppA–heme complex. To explore the DppA conformational flexibility and identify alternative conformations of the protein compatible with heme binding, the 1.27 Å crystallographic model of DppA_Mtb_ was subjected to normal mode analysis. It is well established that low-frequency modes can be useful to describe large-scale motions of proteins^32^. Out of the five lowest frequency modes of DppA, the first three produced a wide opening of the cleft caused by a concerted motion of the two halves that open like a clamp by 10.7 Å, slightly twisting in opposite directions. This predicted opening of DppA halves generates a larger pocket than seen in the crystal structure (Richards’ solvent-accessible volume ~2583 Å^3^), compatible with heme binding (Fig. 5b). Computationally docking heme against the most open conformation predicted by normal mode analysis yielded a physically plausible model (Δ*G* = −7.9 kcal/mole) (Fig. 5c) whereby heme is in bonding distance with several DppA residues including R179, H131, S134, E481, and L477. Specifically, in this model, R179 makes a salt bridge with one of the propionic acid side chains, while H131 is in bonding distance with heme iron (Fig. 5d).

### Heme binding by DppA is essential for Dpp-dependent heme uptake by Mtb

**Fig. 6:**
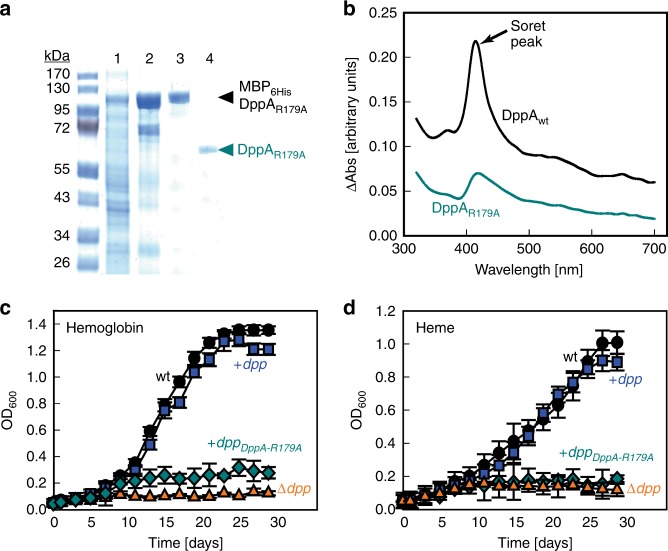
Arginine 179 of DppA is essential for heme binding and utilization by *M. tuberculosis*. **a** Purification of DppA_R179A_ from *E. coli*. Lanes: (1) *E. coli* lysate containing *mbp*_*6his*_*-dppA*_*R179A*_ expression vector, combined fractions after Ni(II)-affinity (2) and amylose affinity (3), (4) purified DppA_R179A_ post TEV cleavage after Ni(II) recapture of MBP_6His_. **b** Difference absorption spectroscopy of DppA_wt_ and DppA_R179A_. The free heme spectra were subtracted from the heme-incubated protein spectra at protein concentrations of 10 µM. Growth of avirulent Mtb mc^2^6206 (circles), the Δ*dpp* mutant ML2436 (triangles), ML2436 complemented with wt *dpp* operon genes (squares) and ML2436 complemented with *dpp* operon genes but expressing *dppA*_*R179A*_ (diamonds). Strains were grown in HdB minimal medium with 2.5 µM human hemoglobin (**c**) or 10 µM hemin (**d**). Medium with hemin and hemoglobin contained 20 µM of 2,2′-dipyridyl to prevent utilization of trace ferric iron. Error bars represent standard errors of mean values of biological triplicates. Please note that the data in Fig. 5c, d for wt (circles), ML2436 (triangles), and ML2436 complemented with wt *dpp* operon genes (squares) are the same as in Fig. 1b. Growth experiments for all strains were performed at the same time. Source data are provided in the Source Data file

To examine the structural model in Fig. 5c, we mutated the amino acids H131 and R179 of DppA_Mtb_ (Fig. 5d). The yield of the DppA_H131A_ protein in *E. coli* was very low, and the purified protein failed to crystallize suggestive of improper protein folding. By contrast, the DppA_R179A_ protein was produced in *E. coli* in similar quantities as wt DppA and was purified to apparent homogeneity with a yield of ~2 mg/l (Fig. 6a). Surface plasmon resonance experiments (Fig. 4b) and difference absorption spectroscopy (Fig. 6b) showed that heme-binding by DppA_R179A_ was almost completely abolished. The DppA_R179A_ protein yielded crystals of excellent quality that were used to determine the 3D-structure to 1.25 Å resolution (Table 1). The atomic structure of DppA_R179A_ is identical to wt DppA with an RMSD ~0.11 Å (Supplementary Fig. 7). The observation that mutation of a single amino acid strongly reduced heme binding by DppA without affecting the protein structure demonstrated the specificity of the interaction. Collectively, these experiments showed that arginine 179 is essential for heme binding and identified the heme binding pocket of DppA_Mtb_. To examine the role of heme binding of DppA, we expressed the *dpp* operon containing wt *dppA* and *dppA*_*R179A*_ in the Mtb Δ*dpp* mutant. While the *dpp* operon fully restored growth, the *dppA*_*R179A*_ gene did not restore growth of the Δ*dpp* mutant with hemin and human hemoglobin as the sole iron sources in HdB minimal medium (Fig. 6c, d). These results showed that under these growth conditions, heme binding by DppA is essential for heme utilization by Mtb.

### Role of tetrapeptide binding in the core of DppA

To examine the effect of tetrapeptide binding by DppA on its affinity for heme, the highly conserved residues W442 and D445 in the peptide-binding pocket of DppA_Mtb_ (Supplementary Fig. 8A) were mutated to alanine. Expression experiments in *E. coli* did not yield any DppA_D445A_ protein suggesting that the mutant protein is misfolded and degraded. By contrast, DppA_W442A_ was purified to apparent homogeneity with a yield of ~1 mg/l (Supplementary Fig. 8B). The DppA_W442A_ protein was monodisperse as determined by size exclusion chromatography (Supplementary Fig. 8B), where it migrated as a monomer similar to wt DppA (Supplementary Fig. 6A). Surface plasmon resonance showed that heme bound to the DppA_W442A_ protein but dissociated quickly (Supplementary Fig. 8C). Screening of thousands of crystallization conditions failed to produce any crystals of DppA_W442A_, in contrast to wt DppA that has a strong tendency to crystallize. These results showed that mutation of tryptophan 442 to alanine reduced the heme binding affinity of DppA, possibly by increasing the dynamic flexibility of the DppA halves, as shown in Fig. 5b.

### The Dpp system is required for Mtb survival in macrophages

**Fig. 7:**
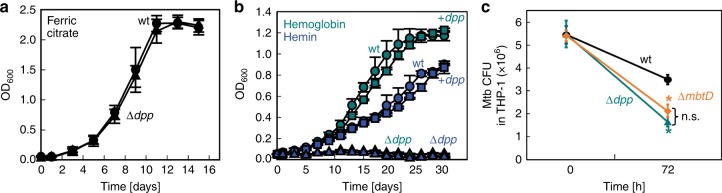
*M. tuberculosis* survival in macrophages depends on heme and iron utilization. Growth of Mtb H37Rv (wt; circles), the Δ*dpp* mutant ML2437 (triangles), and the complemented Δ*dpp* mutant (squares) in HdB minimal medium with **a** 10 µM ammonium ferric citrate and **b** 2.5 µM human hemoglobin (cyan) or 10 µM hemin (blue). **c** THP-1 cells were differentiated with phorbol myristate acetate (PMA) and infected at an MOI of 10 with wt Mtb H37Rv (black) and the Δ*mbtD* (orange) and Δ*dpp* (cyan) mutant strains. Survival of the Mtb strains in macrophages was determined by plating on agar plates and counting colony-forming units (CFU). Asterisks denote significant differences as determined by Tukey’s HSD following an *F*-test (*p* < 0.05) compared to wt. n.s.: not significant. Error bars represent standard errors of mean values of biological triplicates. Source data are provided in the Source Data file

In order to examine the role of the Dpp transporter in virulence of Mtb, we constructed the unmarked Δ*dpp* mutant ML2437 in the virulent Mtb strain H37Rv (Supplementary Table 2). Deletion of the *dpp* operon in Mtb H37Rv completely abolished growth in Hdb minimal medium with hemin and human hemoglobin but did not affect growth with ferric citrate as an iron source (Fig. 7a, b). These results were identical to those obtained before for the avirulent Mtb strains (Fig. 1) and showed that the *ΔpanCD* and the *ΔleuCD* mutations did not have any effect on growth of the *Δdpp* mutant in the avirulent Mtb strain ML2436. Thus, we used the virulent Mtb H37Rv strain and the Δ*dpp* mutant ML2437 to examine whether heme acquisition contributes to Mtb survival in macrophages. To this end, we infected differentiated THP-1 cells with iron-depleted wt Mtb H37Rv, the siderophore-deficient Δ*mbtD* mutant^8^, and the Δ*dpp* mutant. Survival of the Δ*mbtD* and Δ*dpp* mutants was significantly reduced compared to wt Mtb H37Rv 72 h after infection (Fig. 7c).

### Albumin mediates heme and hemoglobin uptake by Mtb

**Fig. 8:**
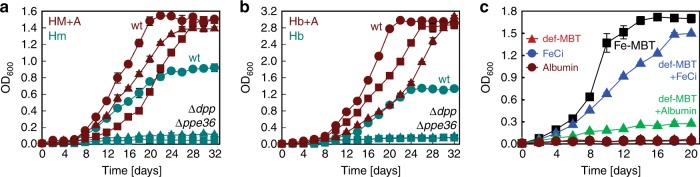
Albumin alters heme and hemoglobin utilization in *M. tuberculosis*. Growth of avirulent Mtb strain mc^2^6206 (wt; circles), Δ*ppe36* (triangles), and Δ*dpp* (squares) in HdB minimal medium without (cyan) or with 0.5% bovine serum albumin (red) containing 10 µM hemin (**a**) or 2.5 µM human hemoglobin (**b**) as sole iron sources. All hemin and hemoglobin media contained 20 µM 2,2′-dipyridyl to prevent utilization of trace ferric iron. Error bars represent standard errors of mean values of biological triplicates. **c** Growth of avirulent Mtb *mbtD* mutant strain ML1600 in HdB minimal medium containing 10 µM ferric mycobactin (Fe-MBT; black squares), 10 µM ferric citrate (FeCi; blue circles), 10 µM FeCi and 10 µM deferrated MBT (def-MBT + FeCi; blue triangles), 10 µM def-MBT (red triangles), 0.5% albumin (green circles) and 10 µM def-MBT and 0.5% albumin (green triangles). Error bars represent standard errors of mean values of biological triplicates. Note: Data points for def-MBT (red triangles), FeCi (blue circles), and albumin (green circles) overlap. Source data are provided in the Source Data file

We made the serendipitous observation that the addition of bovine serum albumin (BSA) to the growth medium significantly alters heme and hemoglobin utilization in Mtb. To examine this effect, we repeated the growth experiments in HdB minimal medium supplemented with 0.5% BSA, the amount in standard Middlebrook 7H9 medium for mycobacteria. Albumin did not affect growth of the Δ*ppe37* mutant in any manner (Fig. 2b). In the absence of albumin, both the *ppe36* and *dpp* mutants did not grow with heme or hemoglobin as the sole iron sources (Fig. 8a, b) consistent with results shown in Fig. 1 and with our previous results^15^. However, albumin stimulated growth of the *ppe36* and *dpp* mutants in medium with heme or hemoglobin to near wild-type levels (Fig. 8a, b) indicating that Mtb bypassed the requirement for PPE36 and the Dpp transporter in the presence of albumin. Inductively coupled plasma mass spectrometry revealed that BSA increased the iron level in HdB medium by fivefold from 13 to 64 µg/l. To assess whether the additional iron in BSA could account for the observed improved growth of Mtb, we utilized the Δ*mbtD* mutant ML1600 which cannot grow in medium containing iron salts unless exogenous siderophores or hemin are added^9^. To this end, we isolated mycobactins from Mtb and removed iron(III) by extensive washing with EDTA. Addition of 10 µM deferrated mycobactin or 10 µM ferric citrate to Hdb minimal medium did not support growth of the Δ*mbtD* mutant (Fig. 8c), in contrast to 10 µM Fe(III)-mycobactin establishing that EDTA-treated MBT does not contain significant amounts of iron. Adding 10 µM ferric citrate to deferrated mycobactin restored growth of the Δ*mbtD* mutant, demonstrating that deferrated mycobactin is capable of mediating iron uptake by Mtb (Fig. 8c). We observed significant growth of the Δ*mbtD* mutant in medium with 0.5% albumin and 10 µM deferrated mycobactin suggesting that mycobactin can sequester albumin-bound iron and mediate its uptake by Mtb. However, growth was much slower compared to mycobactin with 10 µM ferric citrate demonstrating that the iron in albumin only partially contributes to the growth stimulation of the Mtb Δ*ppe36* and Δ*dpp* mutants in medium with heme and albumin (Fig. 8a, b). Altogether, these results show that albumin mediates heme uptake by Mtb mc^2^6206 using an unknown mechanism independent of the Dpp, PPE36 and PPE37 proteins.

## Discussion

Heme is by far the most prevalent iron source in humans^16^. In this study, we showed that the *rv3666c-rv3663c* operon is essential for heme and hemoglobin utilization by Mtb in minimal medium. These genes encode an ABC transporter which also mediates dipeptide uptake in other bacteria^33^. The substrate-binding protein DppA associated with the Dpp transporter binds heme with high affinity. We further showed that arginine 179 is essential for heme-binding by DppA in vitro and for Dpp-dependent heme utilization by Mtb. These results combined with the observations that the Mtb *Δdpp* mutant is more resistant to heme toxicity and experienced iron starvation strongly indicate that the Dpp proteins function as a heme uptake system in Mtb. In previous studies, it was proposed that the inner membrane RND efflux pumps MmpL3 and MmpL11 are involved in heme uptake by Mtb^10,34^. These conclusions were primarily derived from the observations that the Mtb *mmpL11* and the *M. smegmatis mmpL3* mutants grew slower in medium with heme as the sole iron source^10,34^. However, the proposed functions of MmpL3 and MmpL11 in heme uptake based on these experimental observations^10,35,36^ are counterintuitive. MmpL3 and MmpL11 are resistance-nodulation-cell division (RND) efflux pumps involved in export of lipids molecules for biogenesis of the mycobacterial cell wall. While MmpL3 exports trehalose monomycolate^37–39^, MmpL11 transports mycolic acid containing lipids and long-chain triacylglycerolsacross out of the cytoplasm of Mtb^17,40^. Importantly, RND efflux transporter proteins such as the MmpL family proteins function unidirectionally and employ sophisticated mechanisms to utilize the proton motive force to energize export of substrates^41,42^. Thus, the orientation of MmpL proteins in the membrane and the direction of proton flux through these efflux pumps are not compatible with the uptake of substrates. Alternative explanations exist for the observed minor growth defect of the *mmpL3* and *mmpL11* deletion mutants with heme as the only iron source. The *mmpL3* gene is essential in both *M. smegmatis* and Mtb^37^. Thus, it is unlikely to obtain an *mmpL3* deletion mutant in the absence of secondary mutations complicating interpretation of mutant phenotypes^10,34^. Deletion of *mmpL11* alters the lipid composition of the outer membrane and impairs growth of Mtb in vitro^40,43^. It possible that the MmpL3 and MmpL11 efflux pumps play a redundant role in heme detoxification by efflux and, thus, may have a heme-specific utilization defect. In contrast, this study shows that Dpp system is essential for heme uptake by Mtb under our experimental conditions.

The crystal structure of DppA_Mtb_ revealed that a tetrapeptide is bound inside the core at the N- and C-terminal domain interface (Fig. 4c). The peptide-binding site of DppA_Mtb_ is similar to that of other DppA proteins with two highly conserved tryptophan (W442) and aspartate residues (D445) (Supplementary Fig. 8A). Thus, the structural analysis of DppA_Mtb_ is consistent with the function of the Dpp system as a peptide importer as previously deduced from the observation that deletion of the Dpp transporter conferred resistance to the toxic tripeptide bialaphos in Mtb^23^. Since the substrate-binding proteins determine the specificity of their associated ABC transporters^44^, the crystal structure of the DppA_Mtb_-tetrapeptide complex also supports the previous conclusion that the transporter encoded by the *rv3666c-rv3663c* operon is an oligopeptide permease rather than a dipeptide transporter^23^. However, the physiological role of the tetrapeptide is unclear since DppA_Mtb_ was produced without the signal peptide in the cytoplasm of *E. coli*. It is possible that the tetrapeptide bound DppA_Mtb_ in *E. coli* co-translationally, concomitant with the folding of the two halves. The observation that weakening the tetrapeptide binding to DppA with point mutations results in loss, or reduced expression in bacteria supports the idea that the tetrapeptide also plays a structural role inside the DppA core. The apparently tight peptide binding suggests that the interaction of DppA with the DppB/DppC proteins might be needed to transfer the peptide.

DppA_Mtb_ and DppA proteins in other bacteria bind heme (Supplementary Table 4). However, DppA of *E. coli*^45^ and of Mtb, as shown in this study, have been crystallized only with core-bound peptides, while crystal structures of other periplasmic heme-binding proteins associated with ABC-type heme importers such as HmuT show proteins in complex with heme^46,47^. A combination of computational, biochemical, genetic, and physiological approaches enabled us to identify a heme-binding site in DppA_Mtb_, which is located at the surface of DppA_Mtb_ and is separate from the peptide binding site (Figs. 3d and 4 and Supplementary Fig. 9). Our experiments also showed that DppA_Mtb_ is capable of simultaneously binding a tetrapeptide and heme since we purified heme-bound DppA_Mtb_ (Supplementary Fig. 6), but obtained only structures of DppA_Mtb_ with a tetrapeptide but not with heme in different crystal forms (Fig. 4). The inhibition of Dpp-dependent heme utilization in Mtb by tryptone-derived peptides (Supplementary Fig. 5) might be explained by the key role of DppA for the function of the Dpp transporter and competitive binding of tryptone-derived peptides to the heme-binding pocket. The adaptation of the DppA substrate-binding site to accommodate both heme and peptides might also explain the lower heme-binding affinities of DppA proteins (1.5–655 µM) compared to dedicated periplasmic heme-binding proteins such as PhuT and HmuT (1 to 30 nM, Supplementary Table 4). The normal mode analysis indicated that DppA is capable of undergoing conformational changes that open the two protein halves like a clam and enable DppA to bind heme and release it upon binding to the Dpp importer. Since mutations of the conserved tetrapeptide binding residues W442 and D445 destabilized the DppA overall structure and, consequently, heme binding (Supplementary Fig. 8), we propose that the inner tetrapeptide plays a structural role, holding the two halves of DppA together and forming a distal heme-binding pocket, designed for reversible and transient binding of heme.

**Fig. 9:**
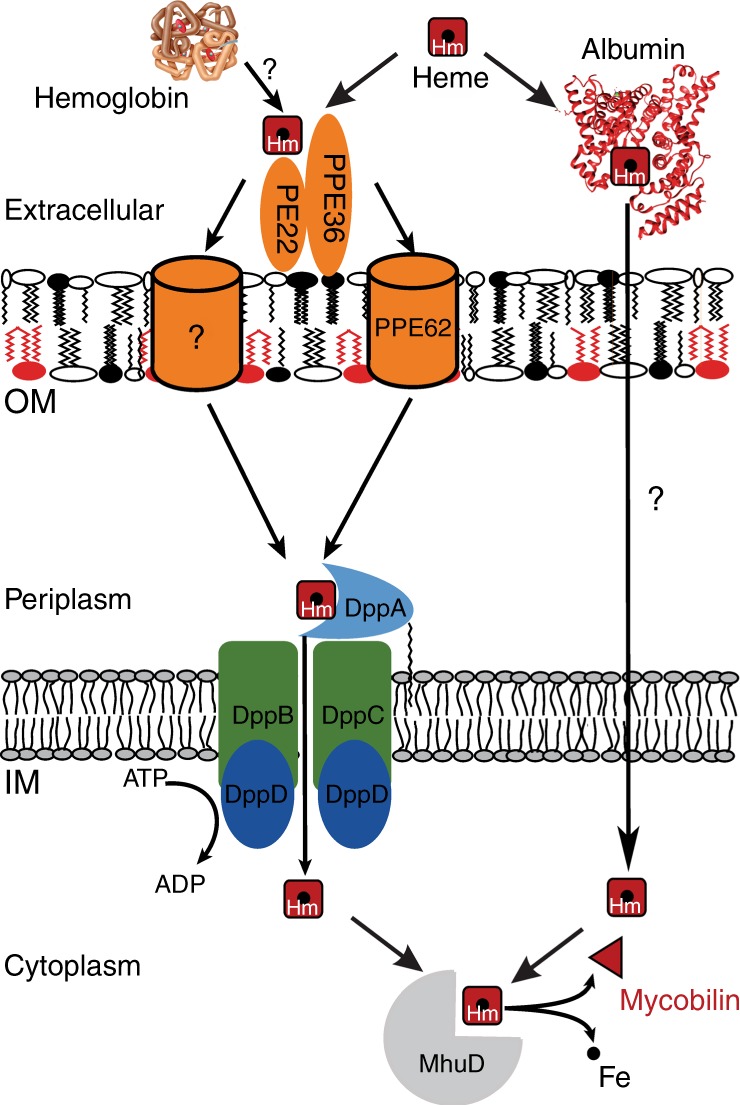
Model of heme acquisition by *M. tuberculosis*. PPE36/PE22 and PPE62 are cell surface-accessible proteins of Mtb that bind heme (red octagon) and are anchored in the outer membrane (OM)^15^. After uptake across the OM, heme is transferred to the periplasmic lipoprotein DppA, possibly with the help of the periplasmic heme-binding protein Rv0265c. DppA probably delivers heme to the DppBCD transporter for uptake across the inner membrane (IM) as shown for other substrate-binding proteins of ABC transporters^75,76^. PPE37, which was recently shown to be essential for heme utilization in Mtb Erdman, is not depicted since its localization is unknown. Cytoplasmic heme is degraded to mycobilin by the oxygenase MhuD thereby releasing iron (black dot). Heme is degraded in the cytoplasm by the oxygenase MhuD to release iron. MmpL3 and MmpL11, which may be involved in heme efflux, are not depicted since it has not been experimentally validated. The fact that PPE36/PE22, PPE62, and the Dpp transporter are required for heme and hemoglobin utilization by Mtb indicates that these pathways converge at the cell surface of Mtb. Our study indicates that there is an alternate heme uptake pathway mediated by albumin, whose components are not known. However, the mechanism by which heme is released from hemoglobin is unknown. Figure adapted from ref. ^15^

Our findings suggest a model of heme and hemoglobin utilization by Mtb (Fig. 9) wherein the surface-exposed, heme-binding membrane proteins PPE36/PE22 and PPE62 are required for both heme and hemoglobin utilization indicating that both pathways converge at the cell surface of Mtb. This implies that heme is stripped from hemoglobin by an unknown mechanism and then transferred to PPE36/PE22 and PPE62. Under our experimental conditions, deletion of PPE62 only partially impairs heme utilization, whereas deletion of PPE36 completely abolished growth of Mtb with heme indicating that it performs an essential function in contrast to PPE62 (ref. ^15^). After crossing the outer membrane, heme is bound by Rv0265c. The growth defect of the Mtb Δ*rv0265c* mutant in heme medium was significant but not severe, indicating that functional homologs of Rv0265c exist in Mtb^15^. Within the periplasm heme is then bound to DppA which possibly transfers heme to the Dpp transporter consisting of the DppB/DppC permease and the DppD ATPase. The function of the substrate-binding protein DppA is essential for heme utilization by Mtb under the experimental conditions in this study. Since we cannot exclude the possibility that DppA may transfer heme to a non-cognate permease, the role of the DppBC permease in heme uptake by Mtb is unclear. The heme uptake mechanism dependent on the Mtb proteins PPE36 and the Dpp transporter in this model (Fig. 9) is different from other pathogenic heme-utilizing bacteria which employ homologs of the *Yersinia enterocolitica* HemTUV permease^14,48^. Our study also revealed an alternative pathway for heme uptake based on albumin, which is independent of the Dpp, PPE36, and PPE37 proteins (Fig. 9). Albumin-mediated heme utilization was also observed in *H. influenzae* strains^49^. Human serum albumin is the most abundant protein in the blood^50^ and specifically binds heme with high affinity^51–53^. BSA is highly similar to human serum albumin (76% homology), is used in mycobacterial media at a concentration of 75 μM and binds hemin with an affinity of 10^4^ 1/M^54^. Thus, at a hemin concentration of 10 μM, as in our experiments, practically all hemin is bound to BSA and not to the lower affinity PPE36 surface protein^15^. The much higher affinity of human serum albumin for hemin with a binding constant of 10 nM^−1^ ^51^ indicates that, during infection of humans, Mtb predominantly uses the albumin pathway for heme uptake when blood is available, e.g., in destroyed lung tissue with extracellular Mtb^55^. In the absence of blood, e.g., when Mtb resides inside cells, the PPE36/DppA pathway is required for heme uptake. Obviously, further experiments are necessary to examine the mechanism of albumin-mediated heme uptake by Mtb. After heme enters the cytoplasm, it is cleaved by the heme oxygenase MhuD releasing iron^36^. Potential heme efflux to reduce the toxicity of excess heme as shown in other bacteria^56^ and as suggested previously for Mtb^15^ is not depicted in this model since it has not been experimentally examined yet.

Recently, it has been shown that PPE37 is essential for heme utilization in the Mtb Erdman strain, but PPE36 is not^24^. We showed in this study that PPE36 is essential for heme utilization in the H37Rv-derived avirulent Mtb strain mc^2^6206, but PPE37 is not. It is possible that genetic variations could result in different requirements for heme utilization between the two Mtb laboratory strains (H37Rv versus Erdman). For example, in our experiments, deletion of *ppe37* stimulated growth of Mtb mc^2^6206 under all conditions tested (Fig. 2). An alternative explanation for the different requirements for PPE36 and PPE37 in the Erdman and H37Rv strains might be the profound effect of albumin on heme utilization by Mtb as observed in our study. While the experiments with the Erdman strain were done in the presence of albumin^24^, our previous^15^ and current experiments with the H37Rv-derived strains were done in medium without albumin. Thus, it is possible that PPE37 is required for uptake of the albumin–hemin complex.

The importance of the *dpp* operon for growth and/or survival of Mtb in vivo was shown previously using an Mtb deletion mutant lacking the *rv3665c-rv3663c* genes. This mutant had a significantly reduced bacterial burden during the chronic phase of the infection in mice compared to wt Mtb^23^. We observed that replication of both the heme-deficient Δ*dpp* and the siderophore-deficient Δ*mbtD* Mtb mutants was impaired in human THP-1 macrophages compared to wt Mtb H37Rv (Fig. 7). Several other genes involved in iron metabolism of Mtb are also required for full virulence of Mtb in mice^7,12,57^. The conclusion that iron availability is important for Mtb during infection is also supported by the observation that high concentrations of transferrin, haptoglobin, and hemopexin are present in the necrotic centers of lung granulomas, the primary sites of infection in human TB patients^5^. These proteins sequester ferric ions, hemoglobin, and heme, respectively, and create an iron-deprived environment. Importantly, iron starvation triggers the transition of Mtb to a persistent, drug-resistant state, while iron availability restarts replication of persistent Mtb^5^. These findings collectively indicate that iron uptake is important for Mtb virulence. Consistent with this conclusion is the emerging complexity of iron acquisition mechanisms in Mtb. Mtb not only produces two classes of siderophores with different biochemical properties, the hydrophobic mycobactins and the more water-soluble carboxymycobactins^58^, but it also employs at least two different mechanisms of iron utilization from heme and hemoglobin as shown in this study and other studies^15,24^. However, it is unclear whether iron acquisition from heme and from iron-siderophore complexes are redundant mechanisms or whether these mechanisms are separately required at different stages of the infection. To evaluate the contribution of these pathways to virulence of Mtb is not trivial because gene deletions in the large mycobactin biosynthesis operon are known to have pleiotropic effects^59^. A similar complication also exists for the Dpp transporter which modulates the transcription of hundreds of genes and alters the lipid composition on the cell surface of Mtb^23^. Further, the dual function of the Dpp transporter makes it difficult to determine whether the in vivo roles of Dpp are due to the lack of heme and/or peptide uptake. In conclusion, the iron-acquisition mechanisms of Mtb are capable of capturing iron from different sources. Their roles in virulence are likely determined by the varying environmental niches of Mtb during infection.

## Methods

### Bacterial strains, media, and growth conditions

Virulent Mtb H37Rv and its derivative strains were grown in Middlebrook liquid 7H9 or solid 7H10 medium supplemented with 10% OADC (8.5 g/l NaCl, 20 g/l dextrose, 50 g/l bovine albumin fraction V, 0.03 g/l catalase, 0.6 ml/l oleic acid). Avirulent Mtb mc^2^6206 and its derivative strains were grown in Middlebrook liquid 7H9 or solid 7H10 medium supplemented with 0.5% glycerol, 10% ADS (8.5 g/l NaCl, 20 g/l dextrose, 50 g/l bovine albumin fraction V), 0.2% casamino acids, 24 μg/ml pantothenate, and 50 µg/ml l-leucine. *Escherichia coli* DH5α was grown in either LB medium containing appropriate antibiotics at 37 °C with shaking at 200 r.p.m. The following antibiotics were used when required: ampicillin (Amp) at 100 μg/ml for *E. coli*, kanamycin (Kan) at 30 μg/ml for mycobacteria, and 50 μg/ml for *E. coli*, and hygromycin (Hyg) at 200 μg/ml for *E. coli* and 50 μg/ml for mycobacteria.

### Targeted gene deletion in *M. tuberculosis*

Gene deletion was performed as previously described^15^. To construct the *dpp* and *ppe37* mutants, upstream (U) and downstream (D) sequences were amplified using corresponding primer pairs UF/SpeI-UR/SwaI and DF/PacI-DR/NsiI (Supplementary Table 4), respectively, and cloned into pML2424 to construct pML3753 (*dpp* deletion vector) and pML3769 (*ppe37* deletion vector) (Supplementary Table 3). A promoter was inserted upstream of *rv3662c* to prevent polar effects from deletion of the *dpp* genes. The deletion vectors were then transformed into avirulent *Mtb* mc^2^6206 and virulent *Mtb* H37Rv. Transformants were selected at 37 °C on 7H10 Hyg and visually validated through the presence of both GFP and RFP fluorescence. Liquid culture of transformant was then plated on 7H10 Hyg containing 2% sucrose at 40 °C for selection of double crossovers. Putative double crossovers were visually analyzed for the presence of only GFP and gene deletion was validated by PCR. For excision of the *loxP*-flanked *gfp*^*2+*^_*m*_*-hyg* cassette, pML2714 expressing Cre recombinase was transformed into marked mutants and unmarked mutants were selected on 7H10 Kan at 37 °C. Putative unmarked mutants were first visually validated through the absence of GFP fluorescence and then through PCR (Supplementary Figs. 1 and 3) (primers, Supplementary Table 4) and loss of growth on hygromycin. The unmarked avirulent and virulent *dpp* mutants were designated as ML2436 and ML2437, respectively, and the unmarked avirulent *ppe37* mutant was designated as ML2451 (Supplementary Table 2).

### Construction of expression vectors for mycobacteria

The *dpp* (*rv3666c-rv3663c*) and *opp* (*rv1283c-rv1280c*) operons were amplified using corresponding primers, 016Clone/F and 016Clone/R (Supplementary Table 4), and cloned into pMN016 to construct pML3757 and pML3758, respectively (Supplementary Table 3). The wild-type copy of *dppA* in pML3757 was replaced with the mutated *dppA*_*R179A*_ gene to construct pML3759 (Supplementary Table 3).

### Growth experiments for determining iron utilization

Strains were first grown in 7H9 medium, then washed in sterile PBS (137 mM NaCl, 2.7 mM KCl, 10 mM Na_2_HPO_4_, 1.8 mM KH_2_PO_4_) with 0.02% Tyloxapol and iron-depleted for 3–4 generations in iron-free 7H9 medium. Strains were then inoculated into HdB minimal medium^60^ containing either 10 µM hemin or 2.5 µM human hemoglobin or 10 µM ammonium ferric citrate as the sole iron source. BSA was added to a final concentration of 0.5% w/v into HdB minimal medium for albumin growth experiments. To prevent growth from iron traces 20 µM of the iron chelator 2,2-dipyridyl (DIP) was added to the medium containing heme or human hemoglobin.

### Production of deferrated mycobactins

Fe(III)-mycobactin (100 mg) was first incubated in the presence of 50 mM EDTA (pH 4.0) at 37 °C for 18 h. EDTA was precipitated by centrifugation and supernatant was collected in a fresh tube. Siderophores were extracted twice by adding an equal volume of chloroform to the supernatant and collecting the top organic layer containing the deferrated mycobactin. Chloroform was removed through evaporation and the deferrated mycobactin residue was suspended in a 1:1 mixture of ethanol and 50 mM KH_2_PO_4_ buffer (pH 7.0).

### Role of *opp* genes and peptides in heme utilization

Strains were first grown to log phase in 7H9 medium, then washed in sterile PBS with 0.02% Tyloxapol and iron-depleted for 3–4 generations in iron-free 7H9 medium. In 96-well plates, washed Mtb cells were inoculated at an OD_600_ of 0.05 into HdB minimal medium^60^ containing either 10 µM hemin or 2.5 µM human hemoglobin or 10 µM ammonium ferric citrate as the sole iron source. For peptide competition assays all media also contained a mixture of tryptone and yeast extract at 1% w/v final concentration. All heme and hemoglobin media contained 20 µM of the iron chelator 2,2-dipyridyl (DIP) to prevent utilization of trace iron. All plates were incubated at 37 °C with shaking for ten days and growth was determined using Alamar Blue assay as described^30^.

### Ethidium bromide accumulation assay

All strains were first grown to log phase in 60 ml of 7H9 medium. Cells were filtered through a 5.0 µM filter to obtain a single-cell suspension and allowed to grow for another 24 h to relieve any membrane stress induced from filtration. Cells were then harvested by low-speed centrifugation at 1500*g* for 10 min and resuspended to a final OD_600_ of 1.0 in uptake buffer (76 mM (NH_4_)_2_SO_4_, 0.5 M KH_2_PO_4_, 1 mM MgSO_4_, 0.4 % glucose and 0.05% Tween-80). For both strains, 100 µl of cells were added in triplicate in a 96-well plate and ethidium bromide was then added to a final concentration of 20 µM. Fluorescence was measured by excitation at 530 nm and emission at 590 nm at 2 min intervals for 60 min.

### Heme toxicity assay

Wild-type H37Rv and mutant derivative strains were first grown to log phase in 7H9 medium, then washed in sterile PBS with 0.02% Tyloxapol and iron-depleted for 3–4 generations in iron-free 7H9 medium. In 96-well plates, washed Mtb cells for all strains were inoculated at an OD_600_ of 0.05 into HdB minimal medium containing 1 µM ammonium ferric citrate and increasing concentrations of hemin. Ammonium ferric citrate was included in the medium to allow consistent growth for all strains. All plates were incubated at 37 °C with shaking for ten days and growth was determined using Alamar Blue assay.

### RNA extraction and quantitative real-time PCR

Strains were first grown to log phase in 7H9 medium, then washed in sterile PBS with 0.02% Tyloxapol and iron-depleted for 3–4 generations in iron-free 7H9 medium. Strains were then inoculated at OD_600_ of 1.0 into 60 ml HdB minimal medium containing either 10 µM hemin or 10 µM ammonium ferric citrate as the sole iron source and incubated for 48 h at 37 °C with shaking at 100 r.p.m. Cells were harvested by centrifugation at 5000*g* for 5 min at 4 °C and resuspended by vortexing in 1 ml Trizol. Cells were lysed with beads at 20 s intervals for 1 min with cooling on ice in between bead beading steps. Beads were precipitated by centrifugation and the Trizol solution was added to 0.3 ml of phenol/chloroform/isoamyl alcohol. Tubes were inverted repeatedly at 30 s intervals for 2 min and then centrifuged at 16,000*g* for 5 min at 4 °C. The aqueous layer was removed to a separate tube containing 0.8 ml of a 1:1 mixture of isopropanol and 3 M sodium acetate. Tubes were inverted several times and nucleic acids were allowed to precipitate overnight at −20 °C. Tubes were then centrifuged at 16,000*g* for 10 min at 4 °C and the nucleic acid pellet was washed twice with ice-cold 70% ethanol. The pellet was air dried and resuspended in 100 µl of DEPC-water. On column DNA digestion and RNA isolation were then performed using the Qiagen RNeasy Kit as per the manufacturer’s protocol. cDNA synthesis was performed using the Bio-Rad iScript cDNA Synthesis Kit as per the manufacturer’s protocol. qRT-PCR cycling conditions for relative quantitation of gene expression was performed using Bio-Rad iQ SYBR Green in a CFX96 Real-Time cycler (Bio-Rad). Cycle threshold (*C*_t_) data were normalized to *rrs* (Mtb 16S rRNA gene) and normalized *C*_t_ values (Δ*C*_t_) were transformed to arbitrary gene expression units using the 2^−Δ*C*t^/10^−6^ method as described by Livak and Schmittgen^61^.

### Construction of expression vectors for *E. coli*

The *dppA* gene, excluding the first 75 base pairs, was amplified using corresponding primers, 1970Clone/F and 1970Clone/R (Supplementary Table 4), and cloned into pML1970 to construct pML3780 (Supplementary Table 3). Mutated DppA_H131A_, DppA_R179A_, DppA_W442A_, and DppA_D445A_ were constructed using mutations primers (Supplementary Table 4) and subsequently validated by Sanger sequencing. The DppA mutants H131A, R179A, W442A, and D445A were cloned into pML1970 to construct pML3781, pML3783, pML3786, and pML3790, respectively (Supplementary Table 3).

### Protein purification

Transformants of pML3780, pML3781, pML3783, and pML3786 in *E. coli* BL21 (DE3) were selected on LB Amp agar plates. All proteins were purified using the same method. Briefly, starter cultures of strains were inoculated into 500 ml of fresh LB medium with ampicillin and grown to an OD_600_ of 0.3. Gene expression was induced with 1 mM IPTG at 18 °C for 24–30 h. After induction of gene expression *E. coli*, cells were harvested by centrifugation and lysed by sonication in ice-cold base buffer (20 mM Tris, 300 mM NaCl, 15% glycerol, 1 mM PMSF, pH 7.4). The cell lysate was clarified by centrifugation and the supernatant was loaded on to activated nickel resin and bound overnight at 4 °C with shaking. Protein loaded resin was washed three times with wash buffer (base buffer with 25 mM imidazole) and target protein was eluted with elution buffer (base buffer with 250 mM imidazole). Eluted protein was then loaded on to activated amylose resin followed by three washes with base buffer and then eluted with base buffer containing 20 mM maltose. The fusion protein was then incubated with purified TEV_6His_ protease (self-made) at room temperature for 24–48 h or until all fusion protein was cleaved. Following cleavage, the protein solution was then loaded on activated nickel resin, where the MBP_6His_ tag and TEV_6His_ were captured on the resin and DppA_Mtb_ was collected in the flow through. Cleaved DppA was further purified using a Superdex 75 (GE Healthcare Life Sciences) size exclusion chromatography column. The final purification yield for DppA_wt_, DppA_R179A_, and DppA_W442A_ were similar (~2 mg/l) while DppA_H131A_ was recovered in small quantities (~0.2 mg/l). All DppA variants were concentrated by ultrafiltration using a Vivaspin20 10 kDa concentrator (Sartorius).

### Crystallization and structure determination

DppA_wt_ and the mutant DppA_R179A_ were crystallized using the vapor diffusion hanging drop method by mixing 2 µl of purified protein (typically concentrated at 15–18 mg/ml) with an equal volume of crystallization solution containing 0.2 M Sodium malonate pH 6.0 and 22% (w/v) PEG3350. In contrast, DppA_H131A_ failed to crystallize. DppA crystals were harvested in nylon cryo-loops, cryo-protected with 27% ethylene glycol and flash-frozen in liquid nitrogen. Diffraction data for DppA_wt_ were collected at beamline 9-2 at a wavelength of 0.97 Å, at Stanford Synchrotron Radiation Lightsource (SSRL), on a Dectris Pilatus 6M detector. Crystals of DppA_R179A_ were diffracted at Cornell High Energy Synchrotron Source (CHESS) F1 beamline station at a wavelength of 0.98 Å, on a Dectris Pilatus 6M detector. All steps of data indexing, integration, and reduction were carried out using HKL2000 (ref. ^62^) and *CCP4* programs^63^. The structure was solved by molecular replacement (MR) using the periplasmic oligopeptide-binding protein from *Salmonella typhimurium* (PDB 1B7H) (~24% sequence identical to DppA_Mtb_) as a search model, as implemented in PHASER^64^. The initial MR solution was refined and partially rebuilt using ARP/wARP^65^, which built approximately 50% of DppA_wt_ residues. This initial model (Rfree ~37%) was then entirely built by alternating cycles of the automated model building with phenix.autobuild^66^ and manual rebuilding using COOT^67^. The completed model was then subjected to positional and anisotropic B-factor refinement in Phenix^68^ and final re-refinement using PDB_redo^69^, which yielded the best *R*_work_/*R*_free_ and stereochemistry. The final models were refined to an *R*_work_/*R*_free_ of 12.8/16.5% (DppA_wt_) and 16.3/18.8% (DppA_R179A_) using all diffraction data between 50–1.27 Å and 15–1.25 Å, respectively. The final DppA_wt_ model has 97.3% of residues in the most favored regions and 2.7% in allowed regions of the Ramachandran plot and no outliers in disallowed regions. DppA_R179A_ has 97.1% of residues in the most favored regions and 2.9% in allowed regions of the Ramachandran plot and no outliers in disallowed regions. Final model validation was done using MolProbity^70^. Crystallographic data collection and refinement statistics are shown in Table 1.

### Structure analysis

Ribbon diagrams and surface representations were prepared using the program Pymol (The PyMOL Molecular Graphics System, Version 2.0 Schrodinger, LLC, https://pymol.org/2/) and Chimera^71^. Intramolecular contacts were measured using PDBsum^72^ and secondary structure superimpositions were carried out in Coot^67^. The webserver CASTp (http://cast.engr.uic.edu)^31^ was used to study DppA surface and identify the volume of solvent-accessible pockets that could be filled by heme. Normal Mode analysis was performed using elNémo webserver (http://www.sciences.univ-nantes.fr/elnemo/index.html)^73^ using three lowest frequency normal modes. elNémo computed ten perturbed atomic models of DppA that are superimposed in Fig. 6b. The lowest frequency with the most open state (state 1) was used for heme docking. Virtual docking was carried out using AutoDock Vina^74^.

### Absorption and surface plasmon resonance spectroscopy

Fresh solutions of hemin were prepared in Tris buffer. An equimolar amount of heme was added to 10 µM apo-DppA_Mtb_ and incubated a room temperature for 5 min. For difference absorption spectroscopy, heme binding was monitored using a Bio-Tek Synergy HT plate reader by subtracting the free heme spectra from the protein-incubated heme spectra. Surface plasmon resonance experiments for detecting heme binding by recombinant 6His-MBP-DppA_Mtb_, 6His-MBP-DppA_R179A_, and 6His-MBP-DppA_W442A_ were performed using a Biacore T200 molecular interaction system (GE Healthcare). HBS-EP (10 mM HEPES, 150 mM NaCl, and 0.005% polysorbate 20 pH 7.4) was used as running buffer for the immobilization and kinetic studies. The recombinant protein was immobilized on a Ni-NTA chip at a flow rate of 5 µl/min for 5 min to obtain a ligand density of ~100 response units (RU). After ligand capture, different concentrations of heme were injected into the flow cell at 10 µl/min for 2 min to observe association, and then dissociation was allowed for 2 min. A reference flow cell without any bound nickel was used for ligand capture to serve as a control. All RU values were normalized to protein capture level and the binding response was reported as a difference between active and control flow cells. A 1:1 binding model was used to fit the response curves with the Biacore evaluation software to calculate the dissociation constant *K*_d_.

### THP-1 macrophage infection

THP-1 monocytes (ATCC TIB-202) were grown in RPMI supplemented with 10% FBS, 10 mM HEPES, 2 mM l-glutamine, 1× non-essential amino acids, 100 U/ml penicillin, 100 µg/ml streptomycin, and 250 ng/ml amphotericin B. The day before infection with Mtb cells, THP-1 monocytes were seeded into 12well plates and differentiated with 50 ng/ml 12-phorbol 13-myristate acetate (PMA). All Mtb strains were first grown in 7H9 medium to mid-log phase (OD_600_ of 0.5), then washed in sterile PBS with 0.02% Tyloxapol and iron-depleted for 3–4 generations in iron-free 7H9 medium. Differentiated macrophages were then infected with Mtb strains at an MOI of 10:1 for 4 h. Following infection, monolayers were incubated with medium containing 20 µg/ml gentamycin for 1 h to kill any extracellular bacteria. Macrophage cells were then lysed at 0 and 72 h post infection and Mtb colony-forming units were enumerated by plating on 7H10 agar.

### Statistical analysis

Sigmaplot (Systat Software) was used for graph development and statistical analysis. Where applicable statistical significance was determined by Tukey’s Honest Significant Difference (HSD) test following an *F*-test. *P* values less than 0.05 are considered significant. All data presented are mean values with error bars representing standard error of mean values of biological triplicates.

### Reporting summary

Further information on research design is available in the Nature Research Reporting Summary linked to this article.

## Supplementary information

Supplementary Information

Reporting Summary

Source Data

## Data Availability

All data presented in this study are available from the corresponding author upon request. The datasets generated during the crystallographic analysis of DppA of *M. tuberculosis* (PDB ID: 6E3D) and of the DppA R179A mutant (PDB ID: 6E4D) are available at the Protein Data Bank at https://www.rcsb.org/. Source data for figures and supplementary figures are provided as a Source Data file. A reporting summary for this article is available as a Supplementary Information file.
