# Supplementary material for: Heme and hemoglobin utilization by Mycobacterium tuberculosis
Source: Nat Commun. 2019 Sep 18;10:4260. doi: 10.1038/s41467-019-12109-5 (PMC6751184; doi:10.1038/s41467-019-12109-5)
Supplement: Supplementary file 1 — Supplementary Information [file 41467_2019_12109_MOESM1_ESM.pdf]

SUPPLEMENTARY INFORMATION

Heme and hemoglobin utilization by *Mycobacterium tuberculosis*

Avishek Mitra[1], Ying-Hui Ko[2], Gino Cingolani[2,3] and Michael Niederweis[1*]

[1] Department of Microbiology, University of Alabama at Birmingham, USA

[2] Department of Biochemistry and Molecular Biology, Thomas Jefferson University, Philadelphia, PA, USA

[3] Institute of Biomembranes and Bioenergetics, National Research Council, Via Amendola 165/A, 70126 Bari, Italy

Running title: Heme and hemoglobin utilization by *M. tuberculosis*

***Address correspondence to:** Michael Niederweis, mnieder@uab.edu

Gino Cingolani, Gino.Cingolani@jefferson.edu

SUPPLEMENTARY FIGURES

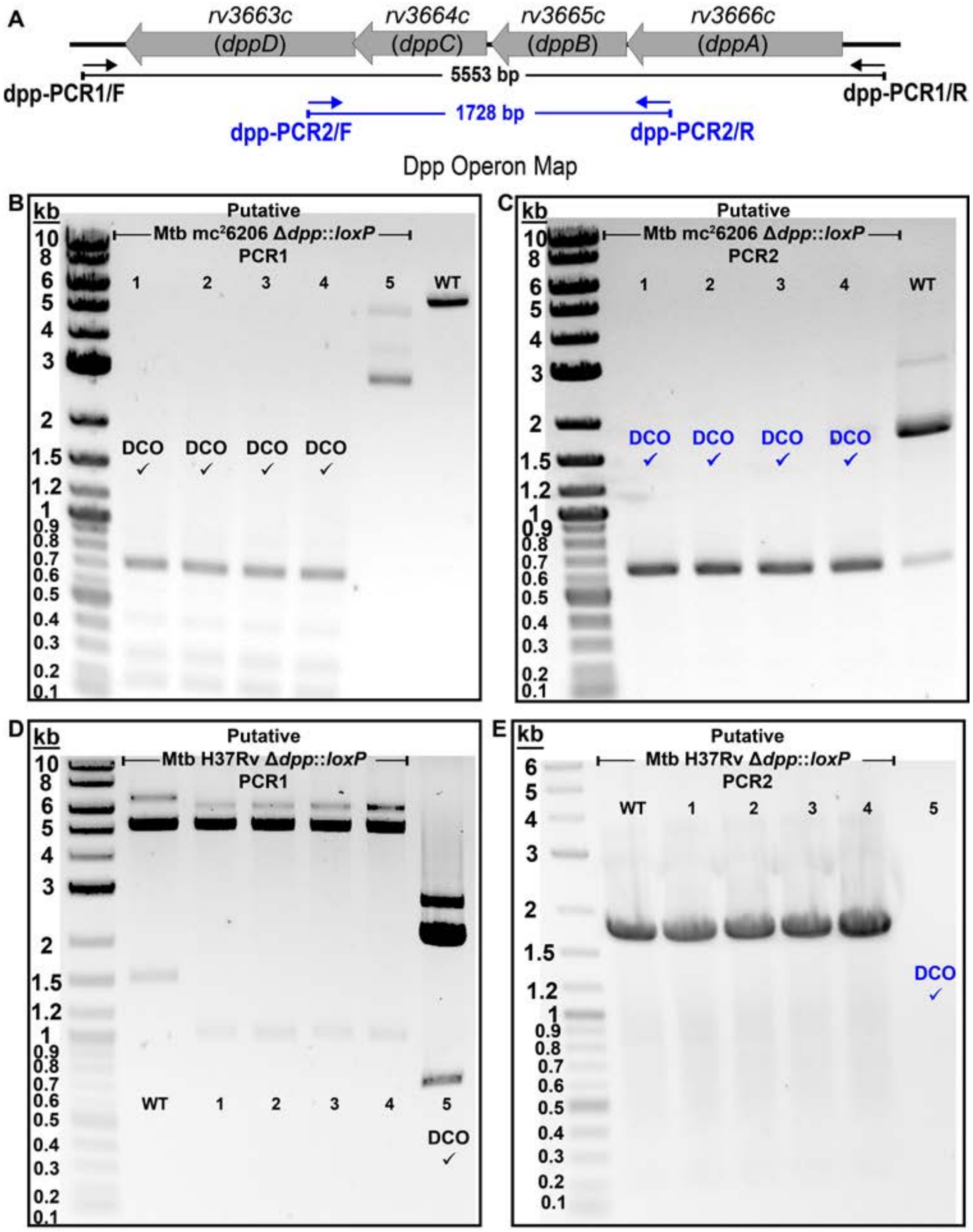

Supplementary Figure 1. Construction of the *M. tuberculosis* Δ*dpp* operon mutant ML2436.

A. Schematic representation of Mtb *dpp* operon and PCRs performed to validate deletion of *dpp* genes. Validation PCR 1 for putative double crossovers in avirulent Mtb mc^{2}6206. **(B)** and virulent Mtb H37Rv **(D)** using primers dpp-PCR1/F and dpp-PCR1/R (external to the *dpp* locus, Table S4). Validation PCR 2 for double crossovers in avirulent Mtb mc^{2}6206 **(C)** and virulent Mtb H37Rv **(E)** using primers dpp-PCR2/F and dpp-PCR2/R (inside of the *dpp* locus, Table S4). Mutant in avirulent and virulent Mtb were denoted ML2436 and ML2437, respectively.

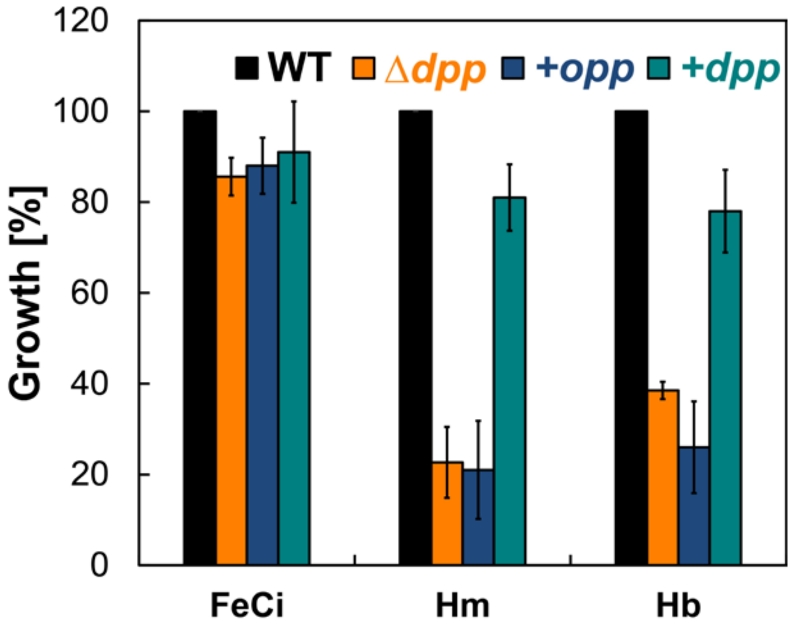

Supplementary Figure 2. The *opp* operon does not complement the heme growth defect of the *M. tuberculosis* Δ*dpp* mutant.

Growth of Mtb mc^{2}6206 (black), the Δ*dpp* mutant (orange) and the Δ*dpp* mutant expressing either the *opp* operon genes (*rv1283c-rv1280c*) (blue) or the *dpp* operon genes (*rv3666c-rv3663c*) (cyan) as measured by the microplate Alamar blue assay. Strains were grown in HdB minimal medium containing 10 µM ammonium ferric citrate (FeCi) or 10 µM hemin or 2.5 µM human hemoglobin. All hemin and hemoglobin medium contained 20 µM of 2,2'-dipyridyl to prevent utilization of trace iron. Error bars represent standard errors of mean values of biological triplicates. Source data are provided in the Source Data file.

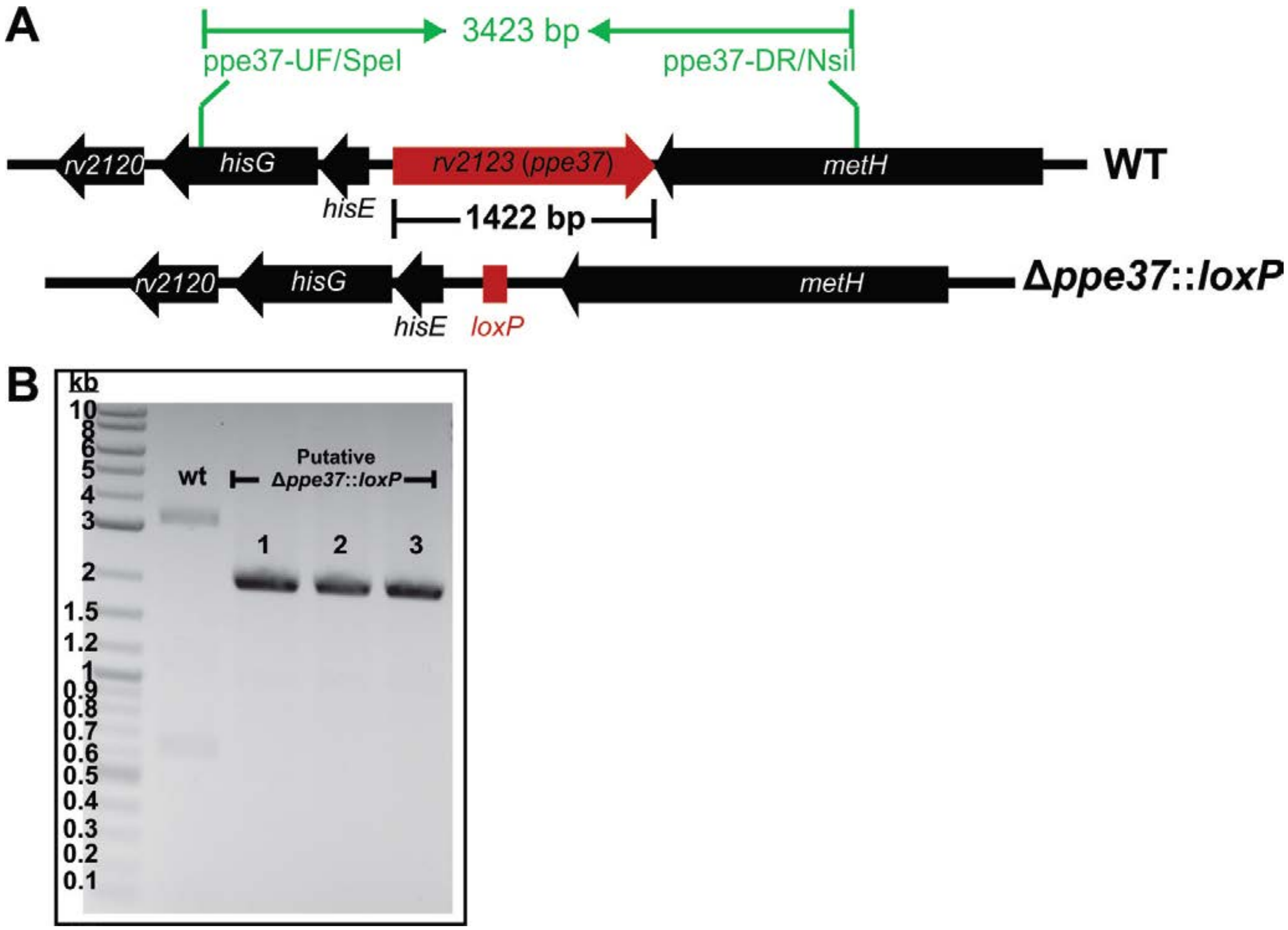

Supplementary Figure 3. Construction of *M. tuberculosis* Δ*ppe37* mutant strain ML2451.

A. Schematic representation of Mtb H37Rv *ppe37* genomic map and PCR performed to validate deletion of *ppe37*. **B.** PCR using primers (Supplementary Table 5) to validate putative unmarked *ppe37* mutants in avirulent Mtb strain mc^{2}6206 (H37Rv Δ*panCD* Δ*leuCD*).

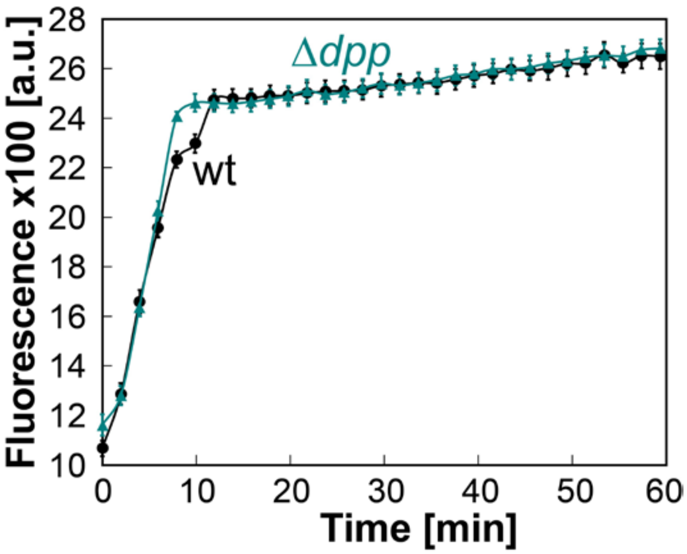

Supplementary Figure 4. Cell permeability of the *M. tuberculosis Δdpp* mutant.

The cell permeability of Mtb mc^{2}6206 (wt, black) and the Δ*dpp* mutant ML2436 (cyan) was determined by measuring ethidium bromide accumulation using fluorescence enhancement upon intercalation of ethidium bromide into DNA as described previously[1]. Error bars represent standard errors of mean values of biological triplicates. Source data are provided in the Source Data file.

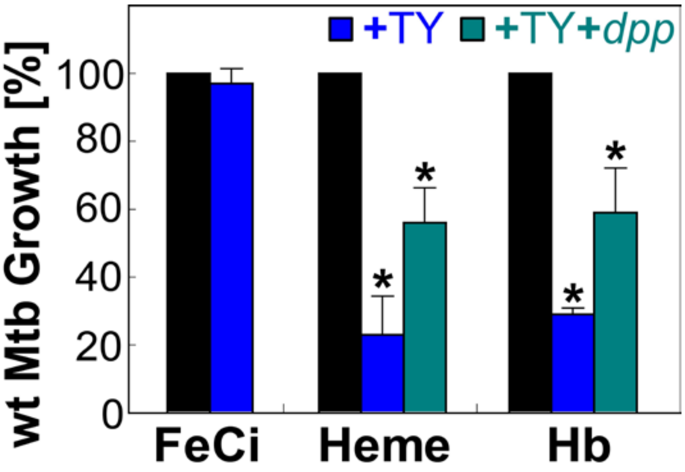

Supplementary Figure 5. Role of peptides in Dpp-dependent heme utilization by *M. tuberculosis*.

Growth of wt Mtb mc^{2}6206 determined using the microplate Alamar Blue assay. Mtb was grown in HdB minimal medium containing different iron sources (10 µM ammonium ferric citrate or 10 µM hemin or 2.5 µM human hemoglobin) [black bars] or in the respective iron sources with 1% TY (tryptone and yeast extract) [blue bars]. Cyan bars show growth of wt Mtb mc^{2}6206 expressing *dpp* genes, in the corresponding HdB medium with TY. All hemin and hemoglobin medium contained 20 µM of 2,2'-dipyridyl to prevent utilization of trace iron. Growth plots with an asterisk are significantly different from growth in medium without TY. Statistical significance was determined by Tukey's HSD following an F-test ($p<0.05$). Error bars represent standard errors of mean values of biological triplicates. Source data are provided in the Source Data file.

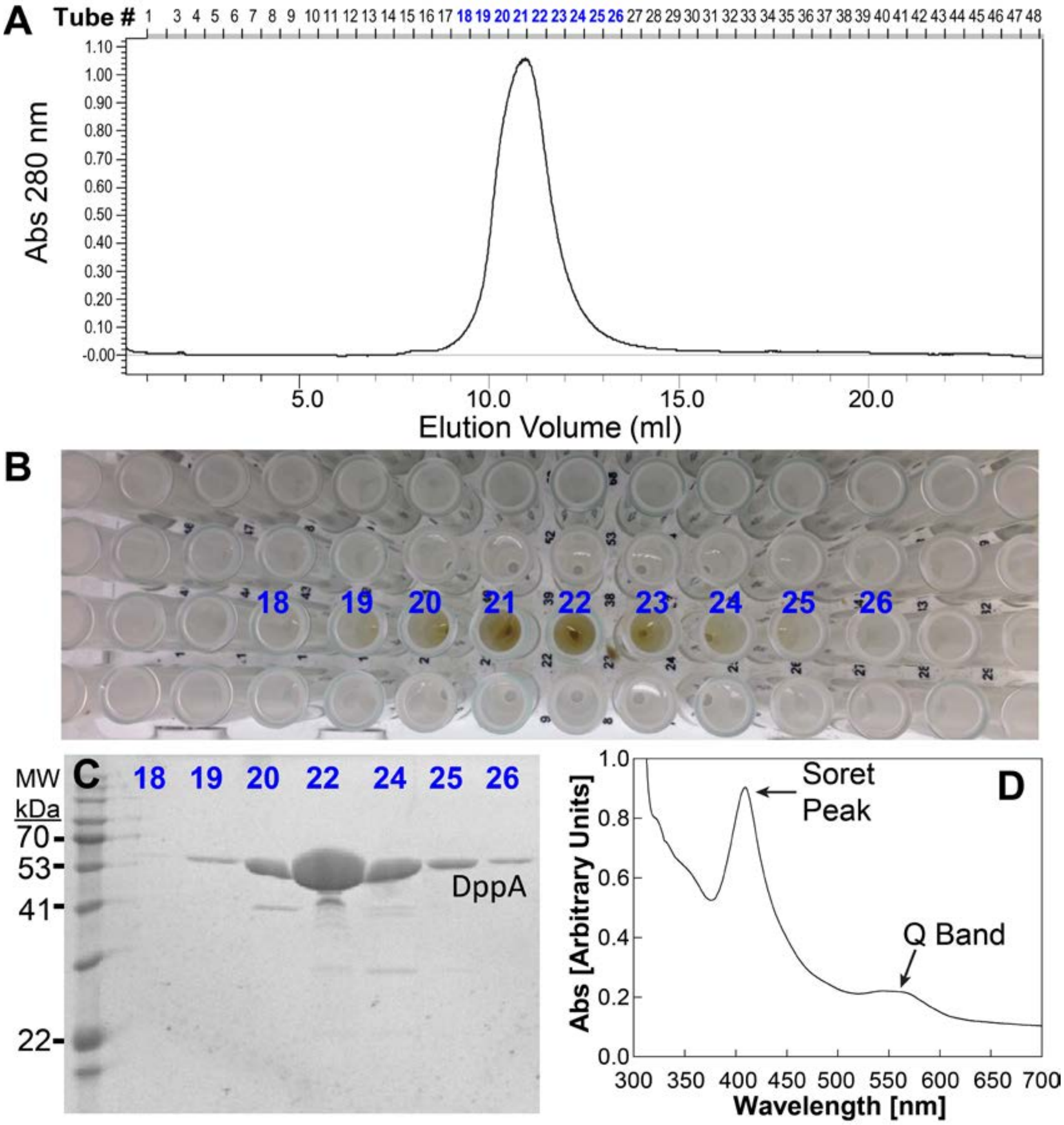

Supplementary Figure 6. Purification of the DppA-heme complex using size exclusion chromatography.

A. The DppA-heme complex was formed in solution by adding a 3-fold molar excess of heme to purified DppA, incubated for one hour at 4 °C and separated on a Superdex 75 column. The eluted fractions #19-25 had a distinct reddish color (**B**) and were resolved by SDS-PAGE (**C**) showing the presence of only DppA. **D.** Absorbance spectrum of the purified DppA-heme complex. A Soret peak at ~405 nm and a broad Q band at ~550 nm are visible. Source data for Figs. 6B and 6C are provided in the Source Data file.

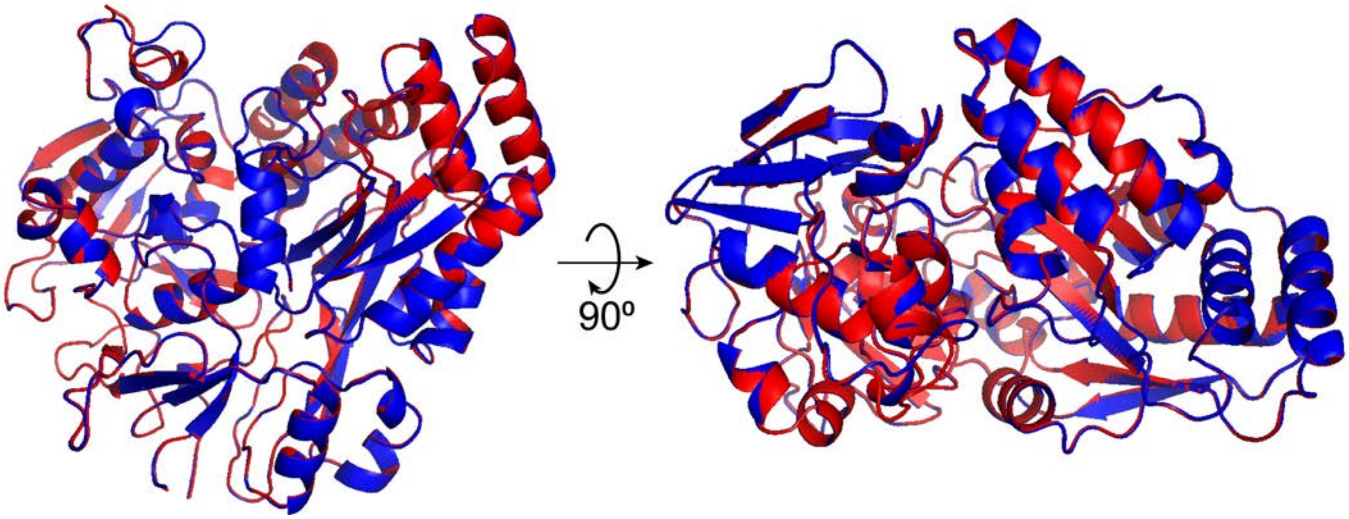

Supplementary Figure 7. Secondary structure superimposition of Mtb DppA$_{wt}$ and DppA$_{R179A}$.

The atomic structures of DppA$_{wt}$ (blue) and DppA$_{R179A}$ (red) were refined to 1.27 and 1.25 Å resolution (**Table 1**), respectively. Secondary structure superimposition suggests the two structures are essentially identical (RMSD ~ 0.11 Å).

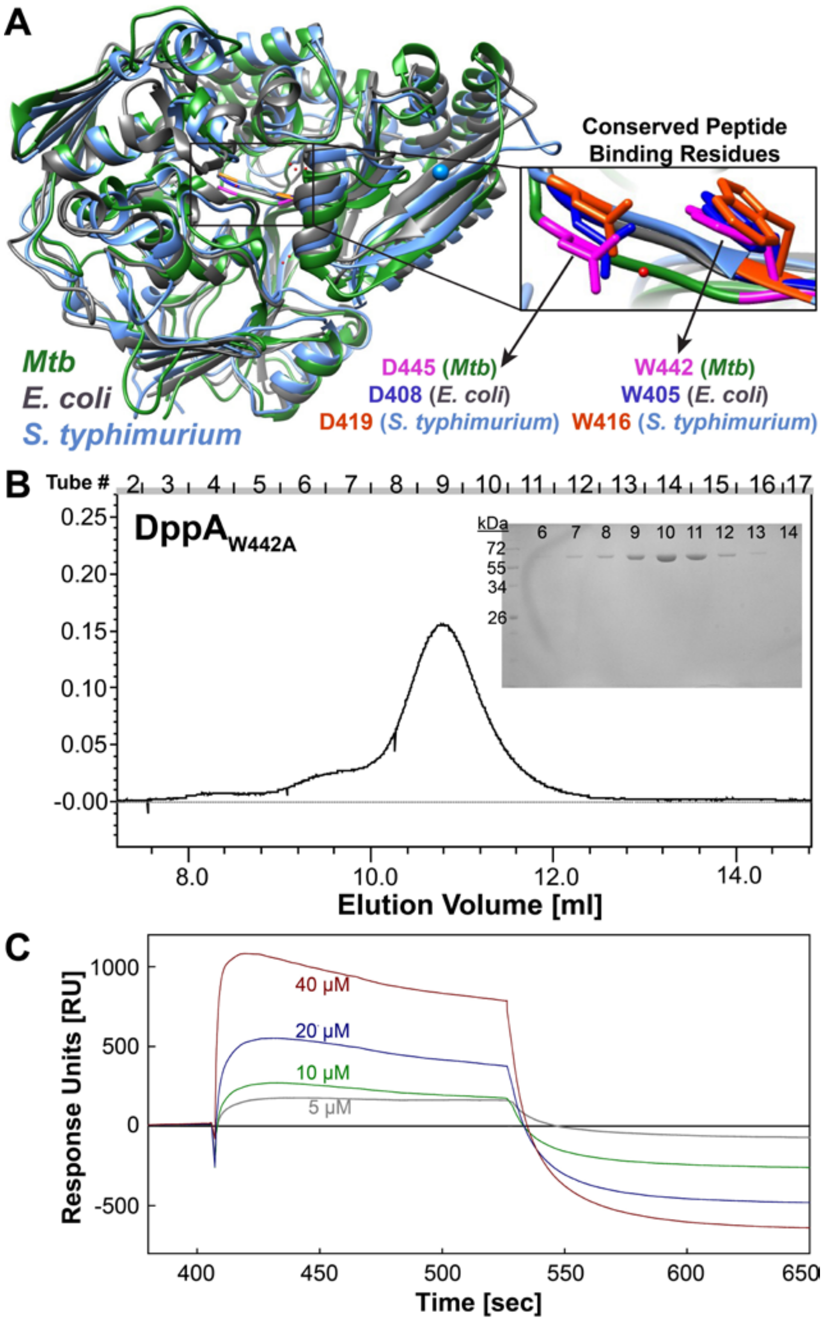

Supplementary Figure 8. Characterization of heme binding by Mtb DppA$_{W442A}$.

A. Secondary structures of DppA$_{Mtb}$ (green) (PDB ID: 6E3D), OppA of *S.typhimurium* (blue) (PDB ID: 1B7H) and DppA$_{Ec}$ (gray) (PDB ID: 1DPP) were superimposed indicating that the core peptide binding pocket is conserved in all proteins. Specifically, two crucial peptide binding residues of DppA$_{Ec}$ (D408, W405) and OppA of *S.typhimurium* (D419, W416) are conserved in DppA$_{Mtb}$ (D445, W442). **B.** Purification of DppA$_{W442A}$ using a Superdex 75 size exclusion chromatography column. **C.** Heme binding of DppA$_{W442A}$ determined by surface plasmon resonance (SPR) spectroscopy.

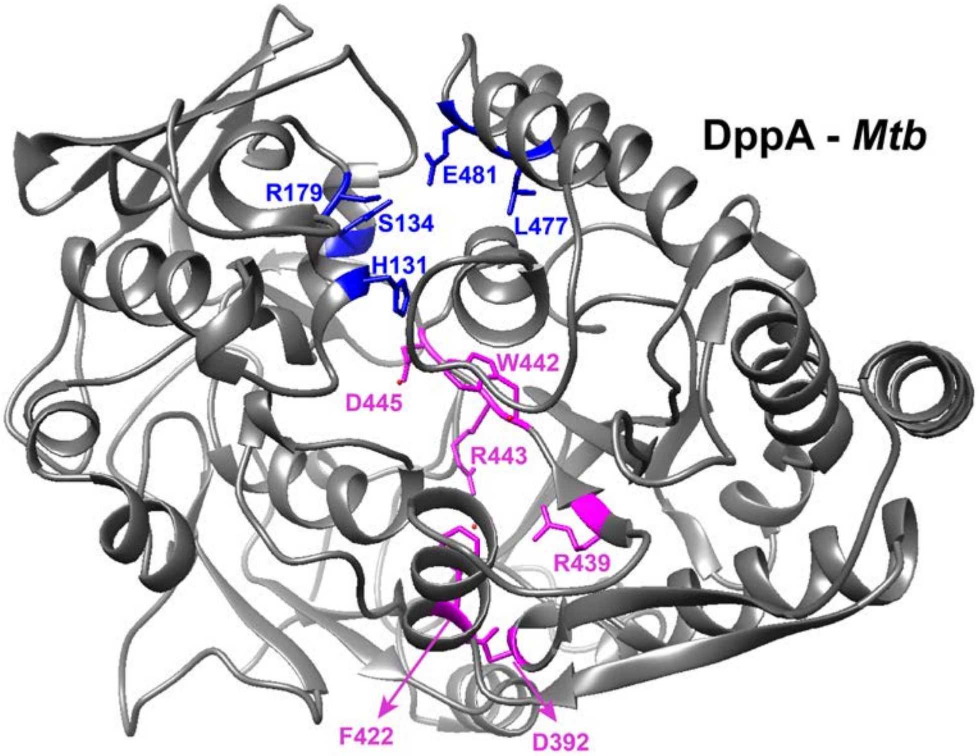

Supplementary Figure 9. Structure of Mtb DppA protein and its substrate binding pockets.

Crystal structure of $DppA_{Mtb}$ (PDB ID: 6E3D) showing the distinct heme (blue) and peptide (magenta) binding pockets.

SUPPLEMENTARY TABLES

Organism	HemTUV or homolog	Growth defect of *hemTUV* mutant	Dpp proteins	Growth defect of *dpp* mutant
Yersinia enterocolitica	+ (HemTUV)	p [2]	+*	n.d.
Vibrio cholerae	+ (HutBCD)	p [3]	+*	n.d.
Pseudomonas aeruginosa	+ (PhuTUV)	m [4]	+[5]	n.d.
Serratia marcescens	+ (HemTUV)	n.d.	+*	n.d.
Shigella	+ (ShuTUV)	n.d.	+*	n.d.
***E. coli* O157:H7**	+ (ChuTUV)	n.d.	+*	n.d.
Neisseria	-	n.a.	+*	n.d.
***E. coli* K12**	-	n.a.	+[6]	ss [6]
Haemophilus influenzae	-	n.a.	+[7]	s to ss [7]
Mycobacterium tuberculosis	-	n.a.	+[8]	ss (This study)

Supplementary Table 1. Distribution and characterization of inner membrane HemTUV and Dpp heme transporters in heme utilizing bacteria.

The growth defect of the respective mutants were examined in medium with heme as the only iron source.

p = partial, m = moderate, s = strong, ss = severe, n.d. = not determined, n.a. = not applicable, * = determined by BLAST analysis.

Genera without species names have homologs in all known species.

Strain	Relevant genotypes and description	Source or reference
E. coli DH5α	*recA1; endA1; gyrA96; thi; relA1; hsdR17(rK-;mK+); supE44; ϕ80ΔlacZΔM15; ΔlacZYA-argF; UE169*	[9]
M. tuberculosis H37Rv	wild-type	ATCC# 25618
M. tuberculosis mc^{2}6206	H37Rv derivative; Δ*leuCD* Δ*panCD*; avirulent *M. tuberculosis*	Dr. Jacobs[10]
M. tuberculosis ML2447	mc^{2}6206 derivative; Δ*dpp*::*hyg* (*rv3666c-rv3663c* deletion mutant; marked)	This study
M. tuberculosis ML2436	mc^{2}6206 derivative; Δ*dpp*::*loxP* (*rv3666c-rv3663c* deletion mutant; unmarked)	This study
M. tuberculosis ML2411	mc^{2}6206 derivative; Δ*ppe36*::*loxP*	[11]
M. tuberculosis ML2412	mc^{2}6206 derivative; Δ*ppe62*::*loxP*	[11]
M. tuberculosis ML1424	H37Rv derivative; Δ*mbtD*::*hyg*	[12]
M. tuberculosis ML2448	H37Rv derivative; Δ*dpp*::*hyg* (*rv3666-rv3663c* deletion mutant; marked)	This study
M. tuberculosis ML2437	H37Rv derivative; Δ*dpp*::*loxP* (*rv3666-rv3663c* deletion mutant; unmarked)	This study
M. tuberculosis ML2451	mc^{2}6206 derivative; Δ*ppe37*::*loxP* (*rv2123* deletion mutant; unmarked)	This study
M. tuberculosis ML1600	mc^{2}6230 derivative; Δ*mbtD*::*hyg* (*rv2381c* deletion mutant; marked)	[13]

Supplementary Table 2. Strains used in this work.
The annotations hyg^R and kan^R indicate that the strain is resistant to the antibiotics hygromycin and kanamycin, respectively. Mutant strains were constructed using either the virulent H37Rv strain or the avirulent mc^{2}6206 strain as parent strains as indicated.

Name	Description	Source
**Parent vectors**		
pML2424	parent vector for construction of deletion mutants in mycobacteria by homologues recombination; Hyg^R	[14]
pML1970	Plasmid used for 6His-MBP-tagged protein purification; Amp^R	[15]
pMN016	cloning vector for expression of mycobacterial genes under strong **psmyc** promoter using PacI-HindIII restriction sites; Hyg^R	[16]
pML2714	*Cre* recombinase vector for excision of *gfp-hyg* cassette utilizing *loxP* sites; Kan^R	[11]
**Knockout vectors**		
pML3753	1000 bp upstream (*Spe*I-*Swa*I) & downstream (*Pac*I-*Nsi*I) of the *dpp* operon (*rv3666c-rv3663c*) cloned into pML2424 (This is the knockout plasmid for deletion of *Mtb dpp* genes). The p_{wmyc} promoter was inserted into the downstream fragment to alleviate any polar effect from *dpp* deletion and allow expression of *rv3662c*; Hyg^R	This study
pML3769	1000 bp upstream (*Spe*I-*Swa*I) & downstream (*Pac*I-*Nsi*I) of *rv2123* (*ppe37*) cloned into pML2424 (This is the knockout plasmid for *rv2123*).	This study
**Expression vectors for mycobacteria**		
pML3757	*dpp* operon (*rv3666c-rv3663c*) cloned into PacI-HindIII digested pMN016 (pMN016 contains strong **psmyc** mycobacterial promoter, can replicate in both *E. coli* and *Mycobacteria*); Hyg^R	This study
pML3758	*opp* operon (*rv1283c-rv1280c*) cloned into PacI-HindIII digested pMN016 (pMN016 contains strong **psmyc** mycobacterial promoter, can replicate in both *E. coli* and *Mycobacterium*); Hyg^R	This study
pML3759	*dpp* operon ($rv3663c_{R179A}$-*rv3666c*) with mutated $dppA_{R179A}$ cloned into PacI-HindIII digested pMN016 (pMN016 contains strong **psmyc** mycobacterial promoter, can replicate in both *E. coli* and *Mycobacteria*); Hyg^R	This study
**Expression vectors for *E. coli***		
pML3780	*dppA* (without first 75 bp encoding signal peptide) cloned into *NdeI-HindIII* digested pML1970 (6His-MBP-tagged DppA for protein purification); Amp^R	This study
pML3781	$dppA_{H131A}$ (without first 75 bp encoding signal peptide) cloned into *NdeI-HindIII* digested pML1970 (6His-MBP-tagged $DppA_{H131A}$ for protein purification); Amp^R	This study
pML3783	$dppA_{R179A}$ (without first 75 bp encoding signal peptide) cloned into *NdeI-HindIII* digested pML1970 (6His-MBP-tagged $DppA_{R179A}$ for protein purification); Amp^R	This study
pML3786	$dppA_{W442A}$ (without first 75 bp encoding signal peptide) cloned into *NdeI-HindIII* digested pML1970 (6His-MBP-tagged $DppA_{R179A}$ for protein purification); Amp^R	This study
pML3790	$dppA_{D445A}$ (without first 75 bp encoding signal peptide) cloned into *NdeI-HindIII* digested pML1970 (6His-MBP-tagged $DppA_{R179A}$ for protein purification); Amp^R	This study

Supplementary Table 3. Plasmids used in this work.
The annotations hyg^R, amp^R and kan^R indicate that the plasmids confer resistance to the antibiotics hygromycin, ampicillin and kanamycin, respectively.

Protein	Organism	Homology	Heme Dissociation Constant (M)	Source/Reference
**DppA**	*Escherichia coli* K12	100%	1×10^{-5}	[6]
**DppA**	*Mycobacterium tuberculosis*	20.8%	1.5×10^{-6}	This study
**HbpA**	*Haemophilus influenzae* Rd	53.1%	6.6×10^{-4}	[17]
**SapA**	Nontypeable *H. influenzae*	28.5%	5.6×10^{-5}	[18]
**ShuT***	*Shigella dysenteriae*	-	$1.1\text{-}5.2 \times 10^{-5}$	[19]
**HmuT**	*Yersinia pestis*	-	3×10^{-8}	[20]
**PhuT**	*Pseudomonas aeruginosa*	-	1.2×10^{-9}	[21]

Supplementary Table 4. Heme binding affinities of known periplasmic substrate binding proteins.
The homology of DppA homologs is indicated as the percentage of similar amino acids compared with *E. coli* DppA.
*The heme dissociation constants were only determined for ShuT mutants, since heme could not be removed from wild-type ShuT.

Name	Sequence	Description
**Gene Deletion & Validation Primers**		
dpp-UF/SpeI	atat**ACTAGT**CGACTCGATCACCGATCTGC	Forward primer for amplifying upstream region of *rv3662c*, contains SpeI restriction site
dpp-UR/SwaI	atat**ATTTAAAT**AACTCCGCGCCGAGGGTGCG	Reverse primer for amplifying upstream region of *rv3662c*, contains SwaI restriction site
dpp-DF/PacI	atat**TTAATTAA**CGGTGAACTTCCAGCCGGGT	Forward primer for amplifying downstream region of *rv3666c*, contains PacI restriction site
dpp-DR/NsiI	atat**ATGCAT**CGCGACGATACAGCCGGCAT	Reverse primer for amplifying downstream region of *rv3666c*, contains NsiI restriction site
dpp-PCR1/F	CCCGGGAGCTCCATTAGCGG	Forward primer for dpp deletion validation PCR1
dpp-PCR1/R	ACTGCACAAGAAGGCTTTAG	Reverse primer for dpp deletion validation PCR1
dpp-PCR2/F	CATGCCCGCGAAGCCGGATC	Forward primer for dpp deletion validation PCR2
dpp-PCR2/R	GGCTGGTATGTTGCGCGCCG	Reverse primer for dpp deletion validation PCR2
ppe37-UF/SpeI	atat**ACTAGT**tcgcgcgcctcggtctggtc	Forward primer for amplifying upstream region of *rv2123*, contains SpeI restriction site
ppe37-UR/SwaI	atat**ATTTAAAT**GTTCGGCTGCCTCCTTCGAC	Reverse primer for amplifying upstream region of *rv2123*, contains SwaI restriction site
ppe37-DF/PacI	atat**TTAATTAA**TGCTTCCGCACACGTGGGAC	Forward primer for amplifying downstream region of *rv2123*, contains PacI restriction site
ppe37-DR/NsiI	atat**ATGCAT**GAAGCCATTAAGGCGCGTGA	Reverse primer for amplifying downstream region of *rv2123*, contains NsiI restriction site
**Complementation & Expression Primers**		
dpp-016Clone/F	atat**TTAATTAA**GGGAGAACAATGGTGCGTCAGATGCGGGC	Forward primer contains PacI restriction site, with optimized RBS sequence from pMN016, 20bp hybridizing to rv3666c, to clone dpp operon into pMN016
dpp-016Clone/R	atat**AAGCTT**TCATCCAGGTGACAACGACT	Reverse primer contains HindIII restriction site, 20bp hybridinzing to rv3662c, to clone dpp operon into pMN016
opp-016Clone/F	atat**TTAATTAA**GGGAGAACAATGACGCGCTATCTGGCCCG	Forward primer contains PacI restriction site, with optimized RBS sequence from pMN016, 20bp hybridizing to rv1283c, to clone opp operon into pMN016
opp-016Clone/R	atat**AAGCTT**TCAGCGTCGCATGAACCCGA	Reverse primer contains HindIII restriction site, 20bp hybridinzing to rv1280c, to clone opp operon into pMN016
**Protein Purification Primers**		
dppA-1970Clone/F	atat**CATATG**TCTTTGAGTTTCGAGGAGGAG	Forward primer contains NdeI restriction site, excludes first 75bp of dppA, to clone dppA into pML1970, to construct pML3780
dppA-1970Clone/R	atat**AAGCTT**AAACTTAGGATGTTCCTTGT	Reverse primer contains HindIII restriction site, to clone dppA into pML1970, to construct pML3780

Supplementary Table 5. Primers used in this work.

Name	Sequence	Description
**Real Time Primers**		
mbtA-RT/F	tattttgctgccgaacgcga	Forward primer for real time PCR of *mbtA*
mbtA-RT/R	atggctcagcagctgttctt	Reverse primer for real time PCR of *mbtA*
16S-RT/F	TGCTACAATGGCCGGTACAAA	Forward primer for real time PCR of *rrs*
16S-RT/F	GCGATTACTAGCGACGCCGACTT	Reverse primer for real time PCR of *rrs*
mbtJ-RT/F	GTGTTGTTTTCGCCGCTGAT	Forward primer for real time PCR of *mbtJ*
mbtJ-RT/R	ATCGACTCCGGTGTAGTACA	Reverse primer for real time PCR of *mbtJ*
mbtB-RT/F	ATGGTCGCCGACATGTTT	Forward primer for real time PCR of *mbtB*
mbtB-RT/R	ATCACGTCGGCACTTGTCA	Reverse primer for real time PCR of *mbtB*
mbtC-RT/F	ATCCACAACCTTGGCGGATT	Forward primer for real time PCR of *mbtC*
mbtC-RT/R	GGTGAGATGCGGAAGAACT	Reverse primer for real time PCR of *mbtC*
mbtL-RT/F	GACGACCTCAACATTGACCT	Forward primer for real time PCR of *mbtL*
mbtL-RT/R	AAGAGCTCCTCTTCGGACAGT	Reverse primer for real time PCR of *mbtL*
mbtM-RT/F	ttaccgctgtaccacgacat	Forward primer for real time PCR of *mbtM*
mbtM-RT/R	gatgaggttgtaggcgaagt	Reverse primer for real time PCR of *mbtM*
**DppA Mutant Primers**		
dppA-H131A	caatgcccaactgcagcag**gcc**tttttcagcccgatcgaag	Point mutation primer for DppA-H131A
dppA-R179A	gaccatcgacttcacgttg**gcc**cttggccacagctcgtttt	Point mutation primer for DppA-R179A
dppA-W442A	agcgagaaactcgatcatcgacgggtagtcgccccgc**gc**tccagcgcgaaatgcactgtc	Point mutation primer for DppA-W442A
dppA-D445A	agcgagaaactcgatcatcgacgggtag**g**cgccccgccatccagcgcgaaatgcactgtc	Point mutation primer for DppA-D445A
dppA-W442A-D445A	agcgagaaactcgatcatcgacgggtag**g**cgccccgc**gc**tccagcgcgaaatgcactgtc	Point mutation primer for DppA-W442A-D445A

Supplementary Table 5. Primers used in this work.

Bold and underlined sequences denote sites for restriction enzymes. Sequences highlighted in red denote point mutations compared to wild-type sequence.

SUPPLEMENTARY REFERENCES

1. Danilchanka O, Mailaender C, Niederweis M. Identification of a novel multidrug efflux pump of *Mycobacterium tuberculosis*. *Antimicrob Agents Chemother* **52**, 2503-2511 (2008).
2. Stojiljkovic I, Hantke K. Transport of haemin across the cytoplasmic membrane through a haemin-specific periplasmic binding-protein-dependent transport system in *Yersinia enterocolitica*. *Mol Microbiol* **13**, 719-732 (1994).
3. Occhino DA, Wyckoff EE, Henderson DP, Wrona TJ, Payne SM. Vibrio cholerae iron transport: haem transport genes are linked to one of two sets of tonB, exbB, exbD genes. *Mol Microbiol* **29**, 1493-1507 (1998).
4. Ochsner UA, Johnson Z, Vasil ML. Genetics and regulation of two distinct haem-uptake systems, phu and has, in Pseudomonas aeruginosa. *Microbiology* **146**, 185-198 (2000).
5. Kiely PD, O'Callaghan J, Abbas A, O'Gara F. Genetic analysis of genes involved in dipeptide metabolism and cytotoxicity in *Pseudomonas aeruginosa* PAO1. *Microbiology* **154**, 2209-2218 (2008).
6. Letoffe S, Delepelaire P, Wandersman C. The housekeeping dipeptide permease is the *Escherichia coli* heme transporter and functions with two optional peptide binding proteins. *Proc Natl Acad Sci U S A* **103**, 12891-12896 (2006).
7. Morton DJ, Seale TW, Vanwagoner TM, Whitby PW, Stull TL. The *dppBCDF* gene cluster of *Haemophilus influenzae*: Role in heme utilization. *BMC Res Notes* **2**, 166 (2009).
8. Flores-Valdez MA, Morris RP, Laval F, Daffe M, Schoolnik GK. *Mycobacterium tuberculosis* modulates its cell surface via an oligopeptide permease (Opp) transport system. *Faseb J* **23**, 4091-4104 (2009).
9. Sambrook J, Fritsch EF, Maniatis T. *Molecular cloning: a laboratory manual*, 2nd edn. Cold Spring Harbor Laboratory Press (1989).
10. Sambandamurthy VK*, et al. Mycobacterium tuberculosis* D*RD1* D*panCD*: a safe and limited replicating mutant strain that protects immunocompetent and immunocompromised mice against experimental tuberculosis. *Vaccine* **24**, 6309-6320 (2006).
11. Mitra A, Speer A, Lin K, Ehrt S, Niederweis M. PPE surface proteins are required for heme utilization by *Mycobacterium tuberculosis*. *MBio* **8**, e01720 (2017).
12. Wells RM*, et al.* Discovery of a siderophore export system essential for virulence of *Mycobacterium tuberculosis*. *PLoS Pathog* **9**, e1003120 (2013).
13. Jones CM, Niederweis M. *Mycobacterium tuberculosis* can utilize heme as an iron source. *J Bacteriol* **193**, 1767-1770 (2011).
14. Ofer N*, et al.* Ectoine biosynthesis in *Mycobacterium smegmatis*. *Appl Environ Microbiol* **78**, 7483-7486 (2012).
15. Pajuelo D, Gonzalez-Juarbe N, Tak U, Sun J, Orihuela CJ, Niederweis M. NAD(+) depletion triggers macrophage necroptosis, a cell death pathway exploited by *Mycobacterium tuberculosis*. *Cell Rep* **24**, 429-440 (2018).
16. Stephan J*, et al.* The growth rate of *Mycobacterium smegmatis* depends on sufficient porin-mediated influx of nutrients. *Mol Microbiol* **58**, 714-730 (2005).
17. Vergauwen B, Elegheert J, Dansercoer A, Devreese B, Savvides SN. Glutathione import in *Haemophilus influenzae* Rd is primed by the periplasmic heme-binding protein HbpA. *Proc Natl Acad Sci U S A* **107**, 13270-13275 (2010).

18. Mason KM, Raffel FK, Ray WC, Bakaletz LO. Heme utilization by nontypeable *Haemophilus influenzae* is essential and dependent on Sap transporter function. *J Bacteriol* **193**, 2527-2535 (2011).

19. Eakanunkul S*, et al.* Characterization of the periplasmic heme-binding protein shut from the heme uptake system of *Shigella dysenteriae*. *Biochemistry* **44**, 13179-13191 (2005).

20. Mattle D, Zeltina A, Woo JS, Goetz BA, Locher KP. Two stacked heme molecules in the binding pocket of the periplasmic heme-binding protein HmuT from *Yersinia pestis*. *J Mol Biol* **404**, 220-231 (2010).

21. Tong Y, Guo M. Cloning and characterization of a novel periplasmic heme-transport protein from the human pathogen *Pseudomonas aeruginosa*. *J Biol Inorg Chem* **12**, 735-750 (2007).
