# Supplementary material for: Heme and hemoglobin utilization by Mycobacterium tuberculosis
Source: Nat Commun. 2019 Sep 18;10:4260. doi: 10.1038/s41467-019-12109-5 (PMC6751184; doi:10.1038/s41467-019-12109-5)
Supplement: Supplementary file 2 — Reporting Summary [file 41467_2019_12109_MOESM2_ESM.pdf]

nature research

Corresponding author(s): Michael Niederweis

Reporting Summary

Nature Research wishes to improve the reproducibility of the work that we publish. This form provides structure for consistency and transparency in reporting. For further information on Nature Research policies, see Authors & Referees and the Editorial Policy Checklist.

Statistical parameters

When statistical analyses are reported, confirm that the following items are present in the relevant location (e.g. figure legend, table legend, main text, or Methods section).

n/a	Confirmed	
☐	☒	The exact sample size (n) for each experimental group/condition, given as a discrete number and unit of measurement
☐	☒	An indication of whether measurements were taken from distinct samples or whether the same sample was measured repeatedly
☐	☒	The statistical test(s) used AND whether they are one- or two-sided<br>*Only common tests should be described solely by name; describe more complex techniques in the Methods section.*
☒	☐	A description of all covariates tested
☒	☐	A description of any assumptions or corrections, such as tests of normality and adjustment for multiple comparisons
☒	☐	A full description of the statistics including central tendency (e.g. means) or other basic estimates (e.g. regression coefficient) AND variation (e.g. standard deviation) or associated estimates of uncertainty (e.g. confidence intervals)
☐	☒	For null hypothesis testing, the test statistic (e.g. F, t, r) with confidence intervals, effect sizes, degrees of freedom and P value noted<br>*Give P values as exact values whenever suitable.*
☒	☐	For Bayesian analysis, information on the choice of priors and Markov chain Monte Carlo settings
☒	☐	For hierarchical and complex designs, identification of the appropriate level for tests and full reporting of outcomes
☒	☐	Estimates of effect sizes (e.g. Cohen's d, Pearson's r), indicating how they were calculated
☐	☒	Clearly defined error bars<br>*State explicitly what error bars represent (e.g. SD, SE, CI)*

Our web collection on statistics for biologists may be useful.

Software and code

Policy information about availability of computer code

Data collection	*Provide a description of all commercial, open source and custom code used to collect the data in this study, specifying the version used OR state that no software was used.*
Data analysis	Sigmaplot (Systat sofware) was used for graph development and statistical analysis.

For manuscripts utilizing custom algorithms or software that are central to the research but not yet described in published literature, software must be made available to editors/reviewers upon request. We strongly encourage code deposition in a community repository (e.g. GitHub). See the Nature Research guidelines for submitting code & software for further information.

Data

Policy information about availability of data

All manuscripts must include a data availability statement. This statement should provide the following information, where applicable:

- Accession codes, unique identifiers, or web links for publicly available datasets
- A list of figures that have associated raw data
- A description of any restrictions on data availability

All data presented in this study are available from the corresponding author upon request. The datasets generated during the crystallographic analysis of DppA of M. tuberculosis (PDB ID: 6E3D) and of the DppA R179A mutant (PDB ID: 6E4D) are available at the Protein Data Bank at https://www.rcsb.org/.

Field-specific reporting

Please select the best fit for your research. If you are not sure, read the appropriate sections before making your selection.

☒ Life sciences ☐ Behavioural & social sciences ☐ Ecological, evolutionary & environmental sciences

For a reference copy of the document with all sections, see nature.com/authors/policies/ReportingSummary-flat.pdf

Life sciences study design

All studies must disclose on these points even when the disclosure is negative.

Sample size	The following process of obtaining biological triplicates was used for all growth experiments, ethidium bromide accumulation assay, RNA extraction, and macrophage infection experiment. For each strain, three single colonies from solid agar plates were separately inoculated into liquid medium providing biological triplicates. Liquid cultures of these biological triplicates were then inoculated into iron-free medium for iron depletion and subsequently into test media or buffer for experimentation.
Data exclusions	No data was excluded
Replication	Absorption spectroscopy for wild-type DppA and mutant variants were performed twice with the same protein sample. The results were consistent both times. This was followed by performing one round of SPR experiments for all proteins.
Randomization	For all growth experiments, ethidium bromide accumulation assay, RNA extraction, and macrophage infection experiment, colonies for all strains were randomly selected from solid agar plates.
Blinding	Our study does not involve any animal or human subjects, as such blinding was not required. Growth and biochemical experiments were performed by AM. For production of DppA protein variants AM constructed mutant genes which were validated by DNA sequencing. Wild type DppA and variants were first purified by AM at UAB and then further purified and validated through crystallization experiments by YK and GC at Thomas Jefferson University. Biochemical experiments determining heme binding by DppA performed at UAB was further validated by purification of heme-DppA complex by YK and GC.

Reporting for specific materials, systems and methods

Materials & experimental systems

n/a	Involved in the study	
☒	☐	Unique biological materials
☒	☐	Antibodies
☐	☒	Eukaryotic cell lines
☒	☐	Palaeontology
☒	☐	Animals and other organisms
☒	☐	Human research participants

Methods

n/a	Involved in the study	
☒	☐	ChIP-seq
☒	☐	Flow cytometry
☒	☐	MRI-based neuroimaging

Eukaryotic cell lines

Policy information about cell lines

Cell line source(s)	THP-1 (ATCC TIB-202)
Authentication	THP-1 cell stocks were received directly from ATCC and further authentication was not performed.
Mycoplasma contamination	THP-1 cells were negative for mycoplasma contamination.
Commonly misidentified lines (See ICLAC register)	None for this study
